# USP32 promotes temporomandibular joint osteoarthritis by modulating PKM2 stability and glycolytic metabolism in chondrocytes

**DOI:** 10.1038/s41419-025-08053-6

**Published:** 2025-11-03

**Authors:** Jiamin Zhao, Runjing Li, Tianjing Du, Mengying Wang, Menghong Li, Zhihui Feng, Zhongbo Liu, Kun Qi

**Affiliations:** 1 Key Laboratory of Shaanxi Province for Craniofacial Precision Medicine Research, College of Stomatology, Xi’an Jiaotong University, Xi’an, China; 2 Department of Orthodontics, College of Stomatology, Xi’an Jiaotong University, Xi’an, China; 3 Department of Geriatrics Cardiology, The Second Affiliated Hospital of Xi’an Jiaotong University, Xi’an, China; 4 Laboratory Center of Stomatology, College of Stomatology, Xi’an Jiaotong University, Xi’an, China; 5 Frontier Institute of Science and Technology, Xi’an Jiaotong University, Xi’an, China; 6 Interdisciplinary Research Center of Frontier Science and Technology, Xi’an Jiaotong University, Xi’an, China

**Keywords:** Deubiquitylating enzymes, Kinases

## Abstract

Metabolic alterations in chondrocytes play a crucial role in the progression of temporomandibular joint osteoarthritis (TMJOA). However, the precise molecular mechanisms underlying these changes remain poorly understood. In this study, we identify ubiquitin-specific protease 32 (USP32) as a key regulator of TMJOA progression through its interaction with pyruvate kinase M2 (PKM2), a vital enzyme in glycolysis. Our results demonstrate that USP32 is significantly upregulated in TMJOA cartilage and inflammatory chondrocytes. USP32 stabilizes PKM2 by removing K48- and K11-linked ubiquitin chains, thereby preventing its proteasomal degradation. This stabilization promotes the accumulation of PKM2, leading to enhanced glycolysis, increased lactate production, and mitochondrial dysfunction, all of which exacerbate chondrocyte apoptosis and the degradation of extracellular matrix. Knocking down USP32 or PKM2 mitigates these detrimental effects, restoring mitochondrial function and reducing inflammation. Furthermore, cartilage-specific knockdown of USP32 alleviates TMJOA pathology in a rat model, highlighting the therapeutic potential of targeting the USP32-PKM2 axis. Our findings reveal a novel mechanism through which USP32 regulates chondrocyte metabolism and inflammation via PKM2 deubiquitination, providing new insights into the pathogenesis of TMJOA and potential therapeutic strategies for its treatment.

## Introduction

Temporomandibular joint osteoarthritis (TMJOA) is a severe form of temporomandibular joint disorder (TMD) that significantly impairs joint function and adversely affects patients’ quality of life. Classified as a degenerative disease, TMJOA affects a substantial portion of the population [1, 2]. Chondrocytes, the sole cellular components of articular cartilage, play a crucial role in the synthesis and maintenance of the cartilage extracellular matrix (ECM), which serves as the primary structural element of cartilage [3]. Consequently, chondrocyte death is a hallmark of cartilage degradation [4]. However, the intrinsic capacity of cartilage for self-repair is severely limited due to its inadequate vascularization and lack of lymphatic drainage, posing significant challenges for effective treatment. Therefore, elucidating the pathological mechanisms underlying condylar degeneration in TMJOA and identifying therapeutic strategies are essential for improving patient outcomes and quality of life.

Ubiquitination regulates post-translational modifications that are critical for cell signaling, fate determination, inflammatory responses, and the degradation of key cellular components essential for various biological processes [5–7]. This process can be reversed by deubiquitination, which is mediated by deubiquitinating enzymes (DUBs) [8]. These enzymes counteract ubiquitination by removing ubiquitin molecules from proteins, thereby regulating numerous cellular mechanisms and maintaining homeostasis [9]. Among the largest families of DUBs, the ubiquitin-specific peptidase (USP) family, USP32 has been implicated in the progression of various diseases, including cancer [10, 11], neurodegenerative disorders [12], regulation of the endosomal transport [13] and autophagy [14]. However, research on the role of USP32 in the context of TMJOA is currently lacking.

In healthy chondrocytes, glycolysis, oxidative phosphorylation, and aerobic glycolysis coexist in a balanced and coordinated manner [15, 16]. Metabolic dysfunction is believed to disrupt the cellular microenvironment and contribute to the progression of osteoarthritis (OA) [17, 18]. Notably, pyruvate kinase M2 (PKM2) has been shown to translocate to the nucleus, where it regulates the expression of various pro-glycolytic enzymes and may play a role in pro-inflammatory processes[19, 20]. Additionally, the transition between PKM2’s dimer and tetramer forms significantly influences mitochondrial function, endoplasmic reticulum stress, and the regulation of other proteins, with the dimer form consistently linked to gene regulation [21].

While extensive research has been conducted on PKM2 in the context of cancer, recent studies have also explored its involvement in OA [22–25]. The functional role of PKM2 in OA remains controversial. It has been shown to exacerbate synovitis by modulating macrophage activity and promoting maturation of pro-IL-1β [26], yet it has also been reported to protect against knee OA by modulating β-catenin signaling pathways [27]. Collectively, the specific mechanistic involvement of PKM2 in TMJOA remains poorly understood, particularly in relation to its regulatory interaction with USP32 and its effect on ubiquitin modification processes. In this study, we present a novel mechanism by which USP32 regulates chondrocyte metabolism and inflammation through modulating PKM2 stability, providing new insights into the pathogenesis of TMJOA and potential avenues for future treatment strategies.

## Materials and methods

### Cell culture and transfection

Primary mandibular condylar chondrocytes (MCCs) were isolated from the temporomandibular joint of 3-week-old male Sprague-Dawley (SD) rats. The cartilage tissue was washed with phosphate-buffered saline (PBS), sectioned into smaller fragments, and subjected to enzymatic digestion using 0.25% trypsin for 30 min, followed by treatment with 2 mg/ml collagenase II for 90 min. The isolated chondrocytes were subsequently filtered through a 40 μm cell strainer and resuspended in Dulbecco’s Modified Eagle Medium-F12 (DMEM-F12), supplemented with 1% antibiotics and 10% fetal bovine serum. Knee cartilage chondrocytes (KCCs) were isolated using the same methodology. Chondrocytes were passaged when reaching approximately 90% confluence. Small interfering RNAs (Supplementary Tables 1, 2, Fig. S8A–E) were transfected to MCCs using Lipofectamine RNAiMAX (Invitrogen, Carlsbad, CA, USA). Adenovirus (AV) overexpression plasmids for USP32 were transfected at a multiplicity of infection (MOI) of 150. Cells within 2 passages were used for subsequent in vitro experiments to prevent dedifferentiation. The ATDC5 mouse chondrocyte cell line was authenticated and cultured in DMEM-F12 medium, supplemented with 1% antibiotics and 10% fetal bovine serum, for verification purposes. Both MCCs and ATDC5 cells were treated with 10 ng/ml of IL-1β (PeproTech, Cranbury, NJ, USA) dissolved in PBS for 24 h.

HEK-293T cells were cultured in DMEM supplemented with 1% antibiotics and 10% fetal bovine serum. Plasmids encoding Flag-USP32, Myc-PKM2, Myc-PKM2 (aa1-375), Myc-PKM2 (aa376-531), HA-Ub-K48, -K11, -K27, -K63, and HA-Ub-WT were transfected into HEK-293T cells using Lipofectamine 2000 (Invitrogen, Carlsbad, CA, USA).

### Animal models

Male SD rats, aged 6–8 weeks, were obtained from the Medical Laboratory Animal Center of Xi’an Jiaotong University, Xi’an, China, for use in the experiments. The rats were randomly assigned to sham and experimental groups. All animal care procedures were approved by the Ethics Committee of Xi’an Jiaotong University (XJTUAE2023-2168) and conducted according to institutional guidelines and the “Animal Research: Reporting In Vivo Experiments” guidelines. Unilateral anterior crossbite (UAC) models were established to build occlusal disorder-induced TMJOA. The UAC and sham groups were conducted as described in the previous study [28]. Sample sizes were calculated using G*Power 3.1 [29], referencing an effect size of Cohen's *d* = 2.92 for apoptosis detection as reported in a previous TMJOA study utilizing UAC models [30], with two-tailed α = 0.10 and power = 0.85, yielding *n* = 3 as statistically adequate. UAC surgeries were performed under sodium pentobarbital anesthesia, with all efforts made to minimize animal suffering. The rats underwent UAC for periods of 4 and 8 weeks to model the establishment and progression of TMJOA, consistent with established protocols in the literature [24, 30–32]. The sham group underwent the same procedure as the UAC group, except for the crown adhesion. Adeno-associated virus (WZ Bioscience Inc., Shandong, China) was used to knock down USP32 specifically in the TMJ chondrocytes by intra-articular injection of pAV-COL2A1-GFP-mir30shUSP32 at a viral dose of 6 × 10^10^ viral genomes per rat. pAV-COL2A1-GFP-mir30shNC was injected as a control. The injection procedure was performed as described in a previous study [33].

### Measurement of cellular bioenergetics by Seahorse assays

Freshly isolated primary chondrocytes were seeded in Seahorse XF96 Cell Culture Microplates (Agilent Technologies, Santa Clara, CA, USA) and cultured in complete medium overnight at 37 °C. After 24 h, the culture medium was replaced with pre-warmed XF base medium (Agilent Technologies, Santa Clara, CA, USA) in preparation for metabolic flux analysis. The cells were then incubated in a 37 °C non-CO2 incubator for 1 h to allow medium equilibration. The microplate was transferred to the Agilent Seahorse XFe96 Analyzer (Agilent Technologies, Santa Clara, CA, USA) for real-time measurement of extracellular acidification rate (ECAR), followed by the Glycolysis Stress Test Kit (Agilent Technologies, Santa Clara, CA, USA), which included 10 mM glucose, 1 mM oligomycin, and 50 mM 2DG [34]. Glycolysis, glycolytic capacity, glycolytic reserve, and non-glycolytic acidification were measured. The measurements were normalized to the total protein content, determined by the bicinchoninic acid protein assay, ensuring accurate quantification and comparability across samples.

### Statistical analysis

Data were expressed as the mean ± SEM or standard deviation and were analyzed using GraphPad Prism (version 9.5, GraphPad Prism Software). All results were generated from a minimum of three independent biological triplicates. The Shapiro-Wilk and Kolmogorov-Smirnov test was performed to evaluate the normality of the data distribution. For non-normally distributed parameters, Mann-Whitney *U* test was utilized for comparisons between two independent groups. Comparisons across multiple groups were conducted using the Kruskal-Wallis test followed by Dunn’s post hoc correction. Otherwise, a two-tailed unpaired *t* test was applied for comparisons between two groups. For comparisons involving multiple samples, a one-way analysis of variance followed by a Bonferroni post hoc test was used. Statistical significance was defined as *P* < 0.05 (95% confidence level).

## Results

### USP32 upregulation exacerbates chondrocyte apoptosis in TMJOA

**Fig. 1:**
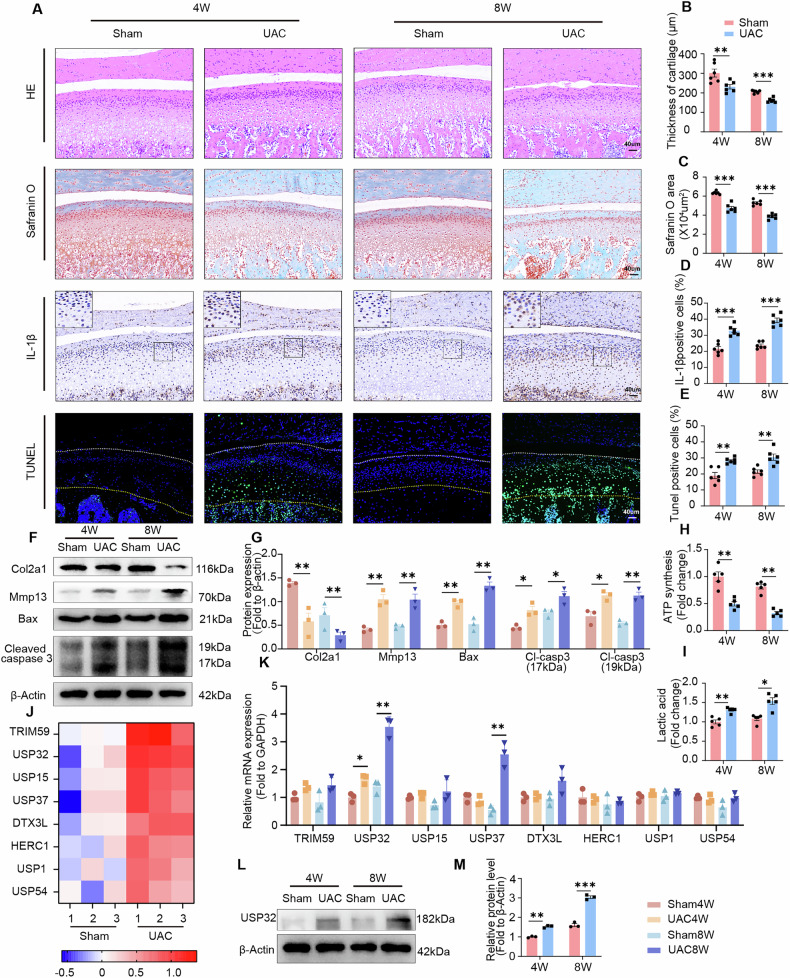
Inflammatory changes in cartilage tissue in the TMJOA model are associated with increased USP32 expression. **A** Histological analysis of central sagittal tissue sections of the condylar cartilage at 4 and 8 weeks from the Sham and UAC groups, including Hematoxylin-eosin (HE) staining, Safranin O staining, IL-1β immunohistochemistry, and TUNEL staining, with the white line representing the cartilage surface and the yellow line marking the junction of chondrocytes and subchondral bone. Scale bar 40 μm. **B–E** Quantitative analysis of histological sections (*N* = 6). **F** Western blot analysis of Col2a1, Mmp13, Bax, and Cleaved caspase 3 protein expression of cartilage tissue. **G** Quantification of Western blot data (*N* = 3). **H** Quantitative evaluation of ATP synthesis in each group (*N* = 5). **I** Quantification of lactate levels in each group (*N* = 5). **J** Heatmap of ubiquitination-related protein expression based on log_2_FC values (GO:0000209, GO:0016579). **K** Quantification of RNA expression levels of the top ubiquitination-related protein in TMJ cartilage (*N* = 3). For *TRIM59*, *USP37* and *HERC1* relative mRNA expression, which showed non-normal distribution, statistical analysis was performed using Mann-Whitney *U* test. **L** Western blot analysis of USP32 expression in TMJ cartilage and quantitative statistical results (*N* = 3) are shown in (**M**). Statistical significance is indicated by *P < 0.05, **P < 0.01, ***P < 0.001.

The UAC model effectively replicates the pathogenesis of TMJOA (Fig. S1A) [30]. Histological analyses revealed progressive cartilage loss, reduced matrix production (Fig. 1A–C), and degeneration of subchondral bone (Fig. S1B–E) over 4 and 8 weeks. Immunohistochemical assessments demonstrated a significant upregulation of IL-1β in chondrocytes (Fig. 1D), accompanied by ECM remodeling, characterized by increased expression of MMP13 and decreased levels of ACAN and Col2a1 (Fig. 1F, G, FisS. 1F–I) [4, 35]. Additionally, there was a marked increase in apoptotic markers, specifically Cleaved caspase 3 and Bax, as well as TUNEL-positive chondrocytes in OA cartilage (Fig. 1E–G). Further analyses indicated a depletion of ATP and an increase in lactic acid (Fig. 1H, I). Mitochondria, which play a crucial role in energy production, exhibited reduced membrane potential, indicating impaired mitochondrial function (Fig. S1J, K). Transcriptome sequencing was performed on cartilage from the UAC and sham groups. All samples exhibited high correlation coefficients (*R*² > 0.926), indicating excellent technical reproducibility (Fig. S2A). Comparative analysis revealed numerous significantly differentially expressed genes (DEGs) between groups (Fig. S2B, D). Notably, these DEGs were enriched in pathways related to metabolic regulation and inflammatory responses (Fig. S2E, F). Among these, DEGs associated with protein deubiquitination (GO:0016579) and ubiquitination (GO:0016567) were significantly enriched (Fig. S2G, H). Consequently, the top eight DEGs (Fold changeå 1.46, FPKMå 1, and *P* < 0.05, Supplementary Table 3) related to protein ubiquitination and deubiquitination were selected. Remarkably, USP32 (Fold change = 2.06) exhibited a substantial increase in cartilage (Fig. 1J, K) and demonstrated consistent time-dependent upregulation (Fig. 1L, M). Collectively, these findings demonstrate that both cartilage and subchondral bone undergo remodeling in TMJOA, attributed to chondrocyte metabolic dysfunction and cell death. The transcriptome sequencing highlights an enrichment in deubiquitination processes, with a notable upregulation of USP32, suggesting its potential role in TMJOA pathogenesis.

### Cartilage-specific knockdown of USP32 alleviates degenerative changes in TMJOA

**Fig. 2:**
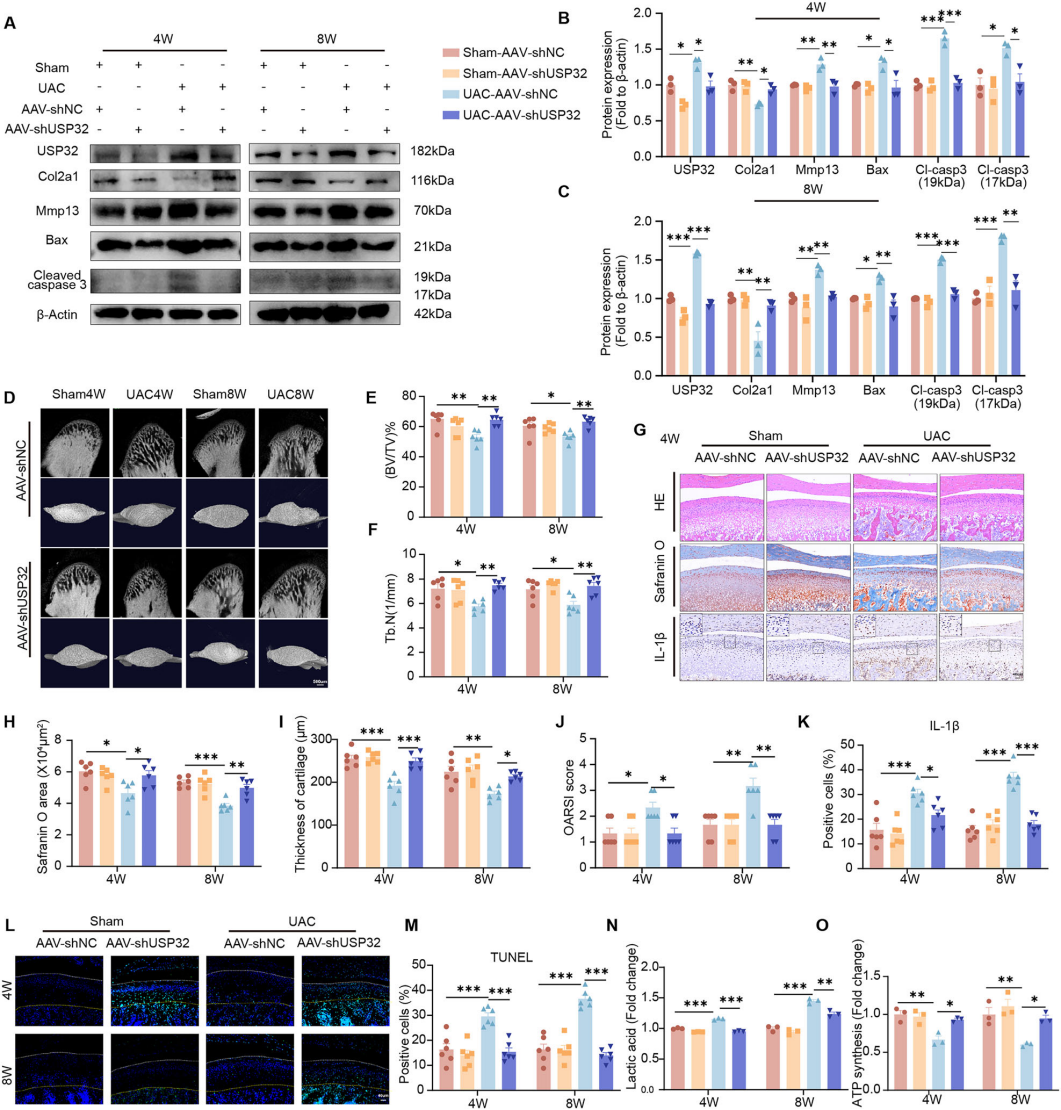
Intra-articular delivery of AAV-shUSP32, specifically knocking down USP32 in articular cartilage, attenuates TMJOA induced by UAC. **A** Protein expression levels of USP32, Col2a1, Mmp13, Bax, and Cleaved caspase 3 in the sham and UAC groups following intra-articular injection of AAV-shUSP32 or AAV-shNC at 4 weeks and 8 weeks. **B** Quantitative analysis of protein expression levels at 4 weeks (*N* = 3). **C** Quantitative analysis of protein expression levels at 8 weeks (*N* = 3). **D** Micro-CT imaging of TMJ and corresponding quantitative analysis of bone volume fraction (BV/TV) shown in (**E**) and trabecular number (Tb.N) shown in (**F**) (*N* = 6). **G** Histological staining of sagittal central sections of the condylar cartilage, including HE staining, Safranin O staining, and immunohistochemical staining for IL-1β (*N* = 6). **H**–**K** Quantitative analysis of histological and immunohistochemical staining results, including Safranin O area, thickness of cartilage, OARSI score, and IL-1β positive cells. **L** TUNEL staining and quantitative analysis of TUNEL-positive cells in each group (**M**); the white line indicates the superficial layer of cartilage, and the yellow line marks the junction between cartilage and subchondral bone (*N* = 6). **N** Quantification of lactate production in each group (*N* = 3). **O** Measurement of ATP synthesis levels across experimental groups (*N* = 3). Statistical significance is indicated by *P < 0.05, **P < 0.01, ***P < 0.001.

In light of the consistent upregulation of USP32 in OA cartilage, we conducted cartilage-specific knockdown of USP32 to elucidate its role in TMJOA. This was achieved using AAV-mediated USP32 knockdown specifically in the cartilage of rats (Fig. S3A–C). Following the knockdown, we observed a significant reduction in apoptosis markers, including Bax and Cleaved caspase 3, as well as a decrease in indicators of ECM degradation, such as Mmp13. Conversely, the expression of Col2a1 was significantly increased at both the 4-week and 8-week time points (Fig. 2A–C). Micro-computed tomography (micro-CT) analysis revealed substantial improvements in the architecture of subchondral bone, indicating enhanced bone quality and structural integrity following USP32 knockdown (Fig. 2D–F). Histological assessment demonstrated improved cartilage preservation, evidenced by increased cartilage thickness, lower Osteoarthritis Research Society International scores [36], and enhanced proteoglycan content (Fig. 2G–J, Fig. S3D). Immunohistochemical analysis confirmed that USP32 knockdown effectively suppressed IL-1β expression (Fig. 2G– K, Fig. S3D).

Additionally, USP32 suppression significantly reduced the number of TUNEL-positive chondrocytes (Fig. 2L, M) and restored metabolic balance, as indicated by increased ATP production and decreased accumulation of lactic acid and pyruvic acid in both the 4-week and 8-week treatment groups (Fig. 2N, O, Fig. S3E). Transmission electron microscopy revealed partial recovery of mitochondrial morphology following USP32 knockdown (Fig. S3F), suggesting an enhancement in bioenergetic function. These findings demonstrate that inhibiting USP32 attenuates OA progression by reducing apoptosis, normalizing metabolic alterations, and maintaining mitochondrial integrity.

### USP32 modulates apoptosis and metabolic changes in inflammatory chondrocytes

**Fig. 3:**
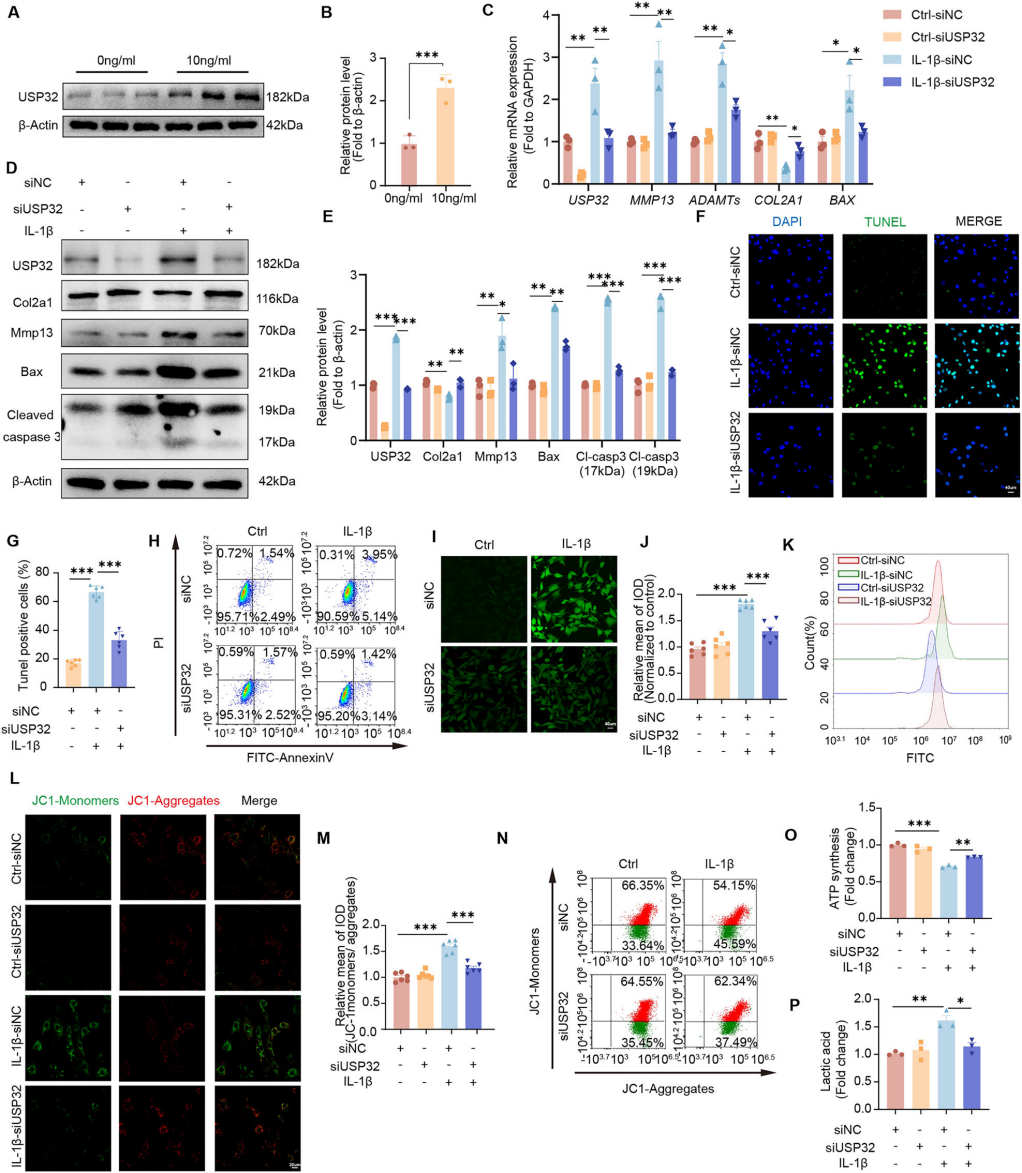
Expression and role of USP32 in inflammatory chondrocytes in vitro. **A** Western blot analysis of USP32 expression in MCCs treated with 10 ng/ml IL-1β or a negative control of PBS and quantitative analysis (*N* = 3) is shown in (**B**). **C** Quantification of RNA expression of *USP32*, *MMP13*, *ADAMTs*, *COL2A1*, and *BAX* (*N* = 3) in siNC and siUSP32-transfected groups under inflammatory and non-inflammatory conditions. **D** Protein expression of USP32, Col2a1, Mmp13, Bax, and Cleaved caspase 3 following transfection of siNC and siUSP32 in each group and quantitative analysis shown in (**E**) (*N* = 3). **F** TUNEL staining of each group, with TUNEL (green) and DAPI (blue) staining, and quantitative analysis shown in (**G**) (*N* = 6). Scale bar 40 μm. **H** Flow cytometric assessment of apoptosis using Annexin V/PI staining in respective groups. **I** Changes in ROS levels in each group and their quantitative analysis in (**J**), along with flow cytometry results (*N* = 6) (**K**). Scale bar 40 μm. **L** JC-1 mitochondrial membrane potential staining in respective groups. Scale bar 20 μm. **M** Quantitative analysis of the JC-1 monomer-to-aggregate fluorescence intensity ratio in each group (*N* = 6). **N** flow cytometry analysis of JC-1 staining. **O** Quantification of ATP synthesis in different groups. **P** Quantitative lactate assay in each treatment group. Statistical significance is indicated by *P < 0.05, **P < 0.01, ***P < 0.001.

Primary MCCs were utilized to examine the role of USP32 in inflammation, apoptosis, and metabolic alterations. Treatment with IL-1β (10 ng/ml for 24 h) significantly induced the upregulation of MMP13, Bax, and Cleaved caspase 3, while concurrently decreasing levels of Col2a1 and ACAN (Fig. S4A–C). This inflammatory response was accompanied by an increase in USP32 expression (Fig. 3A, B). Silencing USP32 via siRNA effectively reversed these effects, leading to a reduction in matrix degradation and apoptosis-related proteins (Fig. 3C–E). Apoptosis assays revealed a higher proportion of apoptotic chondrocytes under inflammatory conditions (Fig. S4D), which was significantly diminished following USP32 knockdown (Fig. 3F–H). Additionally, reactive oxygen species (ROS) levels were elevated in inflammatory chondrocytes (Fig. S4E–H), coinciding with a loss of mitochondrial membrane potential (Fig. S4I–M) with consistent patterns observed in both KCCs and ATDC5 cells, confirming chondrocyte-specific mitochondrial dysfunction. Notably, ROS levels decreased after USP32 knockdown (Fig. 3I–K), which was associated with a restoration of mitochondrial membrane potential (Fig. 3L–N). Furthermore, ATP production showed partial recovery (Fig. 3O), and lactate release was reduced in MCCs that were previously upregulated following inflammatory induction (Fig. 3P, Fig. S4N). These findings suggest that USP32 upregulation in inflammatory chondrocytes is linked to increased apoptosis and metabolic disturbance, while its knockdown confers protective effects.

Moreover, adenoviral overexpression of USP32 in chondrocytes further enhanced the expression of apoptosis-related genes and markers of ECM degradation, while concurrently reducing Col2a1 levels (Fig. S5A–C). The proportion of apoptotic cells significantly increased (Fig. S5D–F), and mitochondrial function and energy metabolism were severely compromised compared to the inflammation-treated group transfected with the control vector (Fig. S5G–L). Furthermore, lactate levels rose, accompanied by a decline in ATP production following USP32 overexpression (Fig. S5M, N). These findings underscore the role of USP32 in exacerbating chondrocyte inflammation, apoptosis, and metabolic dysfunction.

### USP32 interacts with PKM2

**Fig. 4:**
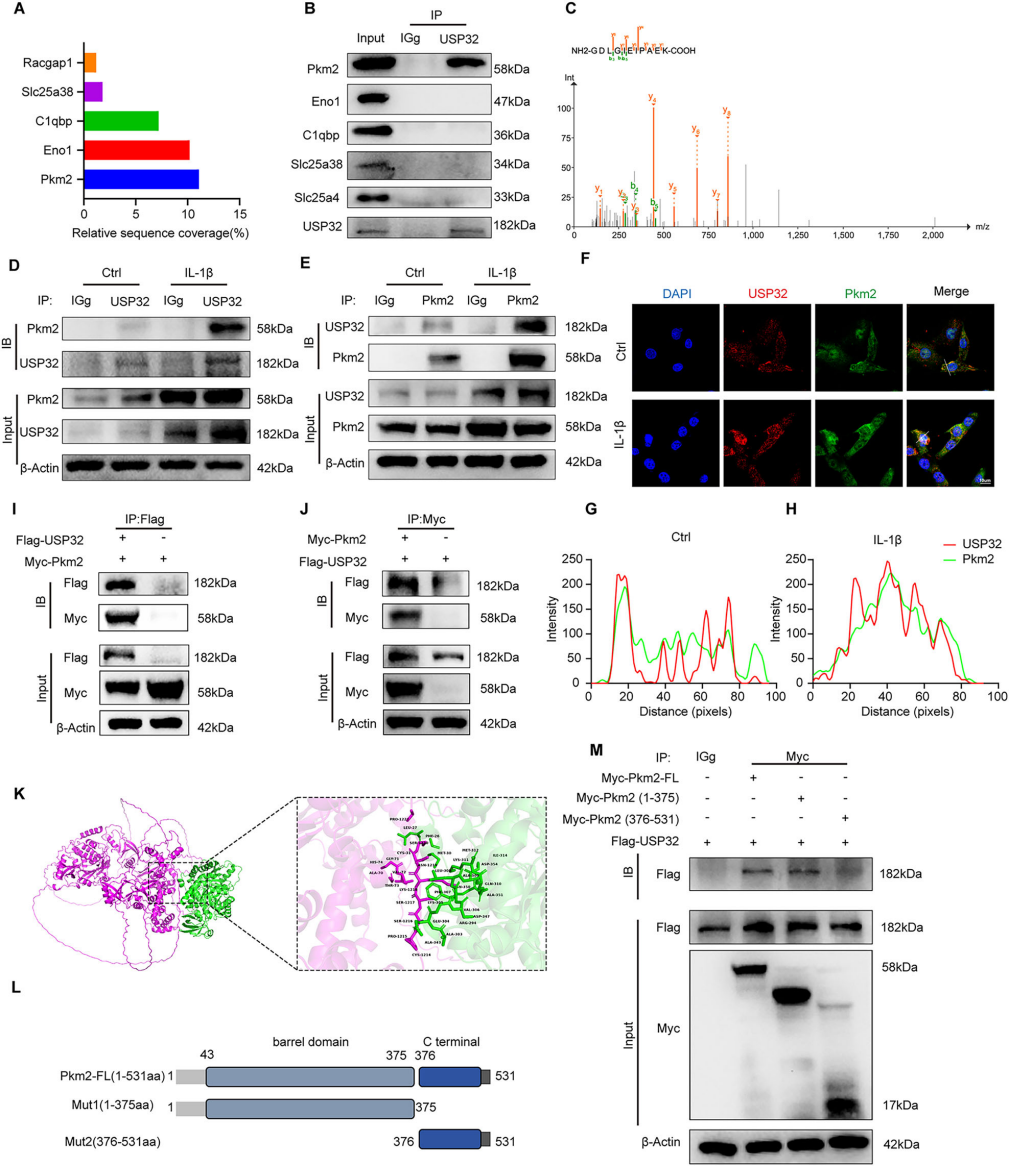
USP32 directly interacts with PKM2. **A** Relative sequence coverage of top metabolic proteins interacting with USP32 detected by immunoprecipitation combined with mass spectrometry. **B** Immunoprecipitation validation of the interaction between USP32 and the top proteins identified in (**A**). **C** Mass spectrometric analysis of immunoprecipitated proteins. **D** Co-immunoprecipitation of USP32 and PKM2 in MCCs under inflammatory and normal conditions using anti-USP32 antibody and immunoglobulin G (IgG). **E** Co-immunoprecipitation of USP32 and PKM2 in MCCs under inflammatory and normal conditions using anti-PKM2 antibody and IgG. **F** Immunofluorescence staining of USP32 (red) and PKM2 (green) in MCCs; the quantification of fluorescence intensity was based on the white line in the control group (**G**) and IL-1β group (**H**). Scale bar 10 μm. **I** Coimmunoprecipitation of USP32 and PKM2 in HEK-293T cells co-transfected with Flag-USP32 and Myc-PKM2 overexpression plasmids. USP32 was immunoprecipitated by anti-Flag antibody. **J** PKM2 was immunoprecipitated by anti-Myc antibody. **K** AlphaFold prediction of the molecular docking between USP32 and PKM2. **L** The schematic of the PKM2 truncated mutation is shown on the left. **M** Immunoprecipitation of Flag-USP32 and Myc-tagged PKM2 truncated mutant or full-length plasmids co-transfected into HEK-293T cells using anti-Myc antibody.

As a DUB, USP32 plays a critical role in modulating the function and stability of substrate proteins by regulating their ubiquitination status. Through co-immunoprecipitation (Co-IP) and liquid chromatography-mass spectrometry (LC-MS), we identified five key metabolism-related proteins that interact with USP32 (Fig. 4A, Fig. S6A, Supplementary Table 4). Notably, PKM2, a vital glycolytic enzyme [37, 38], exhibited a stable interaction with USP32 (Fig. 4B, C). We confirmed the interaction between USP32 and PKM2 in MCCs, with inflammatory stimulation enhancing their binding, as demonstrated by Co-IP and immunofluorescence analysis (Fig. 4D–H, Fig. S6B). Additionally, co-transfection experiments in HEK-293T cells using Flag-USP32 and Myc-PKM2 overexpression plasmids further substantiated this direct interaction (Fig. 4I, J).

To delineate the interaction domain, AlphaFold predictions indicated potential binding sites within USP32, specifically in the amino acids around 66-76 and 1200-1250, and in PKM2, around amino acids 25-31, 131-136, and 291-401 (Fig. 4K, Fig. S6C) [39]. We co-transfected HEK-293T cells with Myc-tagged mutants of PKM2, targeting the barrel domain and C-terminal, alongside Flag-tagged USP32 plasmids. The IP results indicated that USP32 specifically binds to PKM2’s barrel domain (aa 1-375) (Fig. 4L, M). These findings confirm that USP32 directly interacts with PKM2 through its barrel domain.

### USP32 stabilizes PKM2 via deubiquitination

**Fig. 5:**
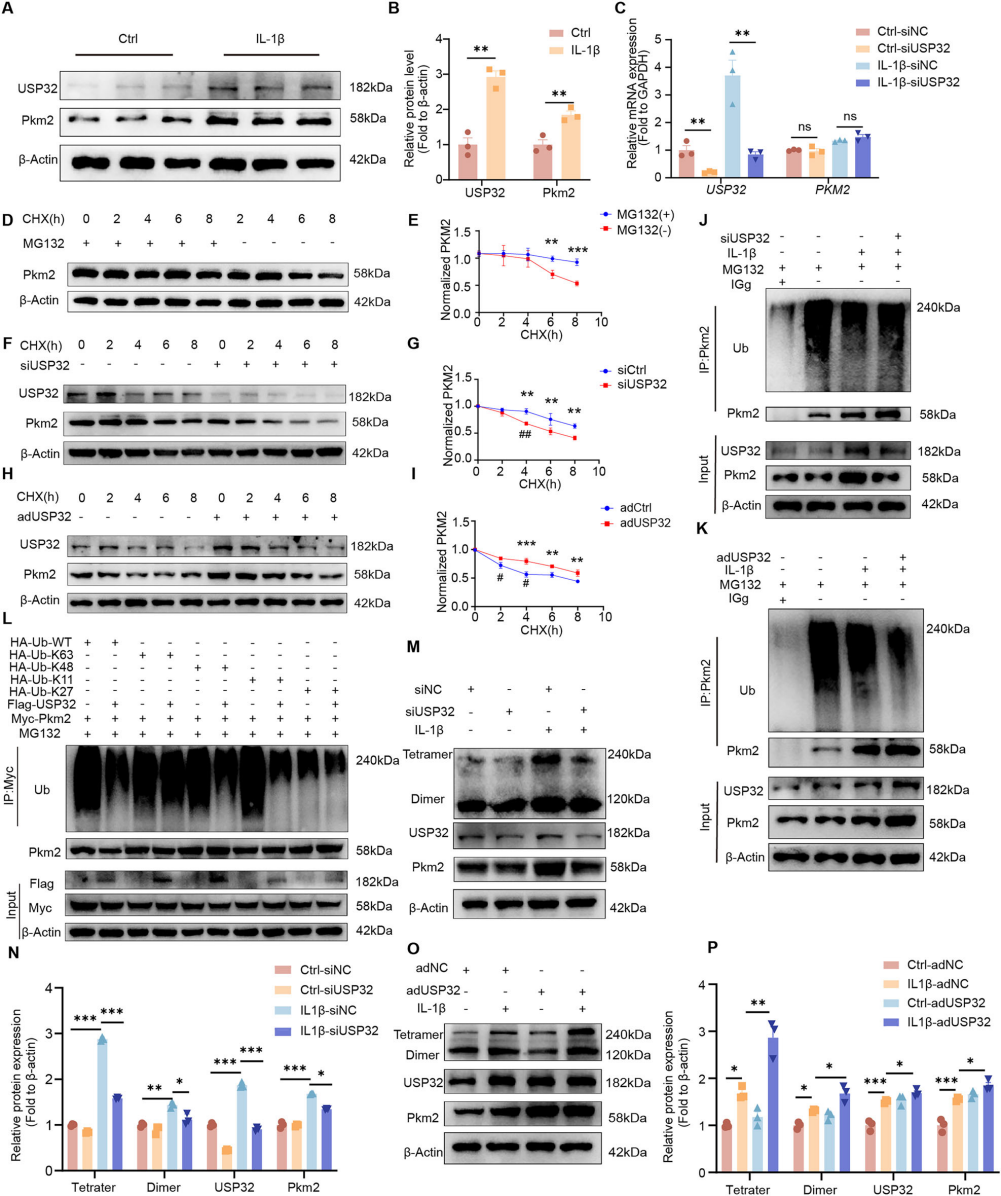
USP32 regulates PKM2 protein stability through deubiquitination. **A** Protein expression of USP32 and PKM2 in MCCs in the control and inflammatory groups, with quantification shown in (**B**). **C** RNA expression levels of *USP32* and *PKM2* in MCCs transfected with siNC or siUSP32 under normal and inflammatory conditions by RT-qPCR. **D** Time-dependent changes in PKM2 protein expression in the presence of MG132(10 μM) and vehicle, following treatment with CHX (50 μM), with quantification on the right (**E**). **F** Time-dependent changes in PKM2 protein expression in MCCs transfected with siNC or siUSP32 in the presence of CHX, with quantification on the right (**G**). **H** Time-dependent changes in PKM2 protein expression in MCCs transfected with adNC or adUSP32 in the presence of CHX, with quantification on the right (**I**) where * indicates significant differences in relative protein levels between groups at specific time points and # represents significant differences in degradation rates during the 2-h intervals preceding each time point. **J** After the transfection of siNC or siUSP32 in the inflammatory or control group in MCCs, MG132 was used to inhibit proteasomal degradation, followed by immunoprecipitation with anti-PKM2 antibody to study changes in ubiquitin levels. The same analysis was performed in cells transfected with adNC or adUSP32 (**K**). **L** Immunoprecipitation of PKM2 in HEK-293T cells transfected with Flag-USP32 overexpression or vector control plasmids that were co-transfected with Myc-PKM2, and HA-Ub-WT, HA-Ub-K48, HA-Ub-K11, HA-Ub-K27, or HA-Ub-K63 overexpression plasmids. Following MG132 treatment (10 μM). Immunoprecipitation with anti-Myc antibody was used to detect changes in ubiquitination levels. **M** Expression of USP32 and PKM2, and its dimers and tetramers in MCCs transfected with siNC or siUSP32, with quantification shown below (**N**). **O** Expression of USP32 and PKM2, and its dimers and tetramers in cells transfected with adNC or adUSP32, with quantification shown on the right (**P**). Statistical significance is indicated by*/# *P* < 0.05, **/## *P* < 0.01, *** *P* < 0.001.

The regulatory mechanism underlying the interaction between USP32 and PKM2 was further investigated. Inflammatory stimulation resulted in increased protein levels of both USP32 and PKM2 (Fig. 5A, B). Notably, silencing USP32 did not significantly affect the RNA expression levels of PKM2 (Fig. 5C). Cycloheximide chase assays, combined with MG132 treatment, demonstrated that USP32 reduces the degradation rate of PKM2 protein by inhibiting its proteasomal degradation (Fig. 5D–I). Inflammatory conditions were associated with decreased ubiquitination of PKM2, which corresponded with an increase in its protein expression. Conversely, silencing USP32 led to increased ubiquitination of PKM2 and a reduction in its protein levels, while overexpression of USP32 resulted in the opposite effect (Fig. 5J, K). Ubiquitin linkage analysis in HEK-293T cells confirmed that USP32 regulates PKM2 by cleaving K48- and K11-linked ubiquitin chains (Fig. 5L). Moreover, USP32 markedly stabilized both tetrameric and dimeric forms of PKM2 in MCCs, with a more pronounced effect on the tetrameric conformation. (Fig. 5M–P). These findings illustrate that USP32 functions as a post-translational regulator of PKM2 by cleaving K48- and K11-linked ubiquitin chains, thereby preventing its proteasomal degradation and notably enhancing the stability of its tetrameric form.

### Glycolytic metabolism via the USP32-PKM2 axis drives inflammatory responses in chondrocytes

**Fig. 6:**
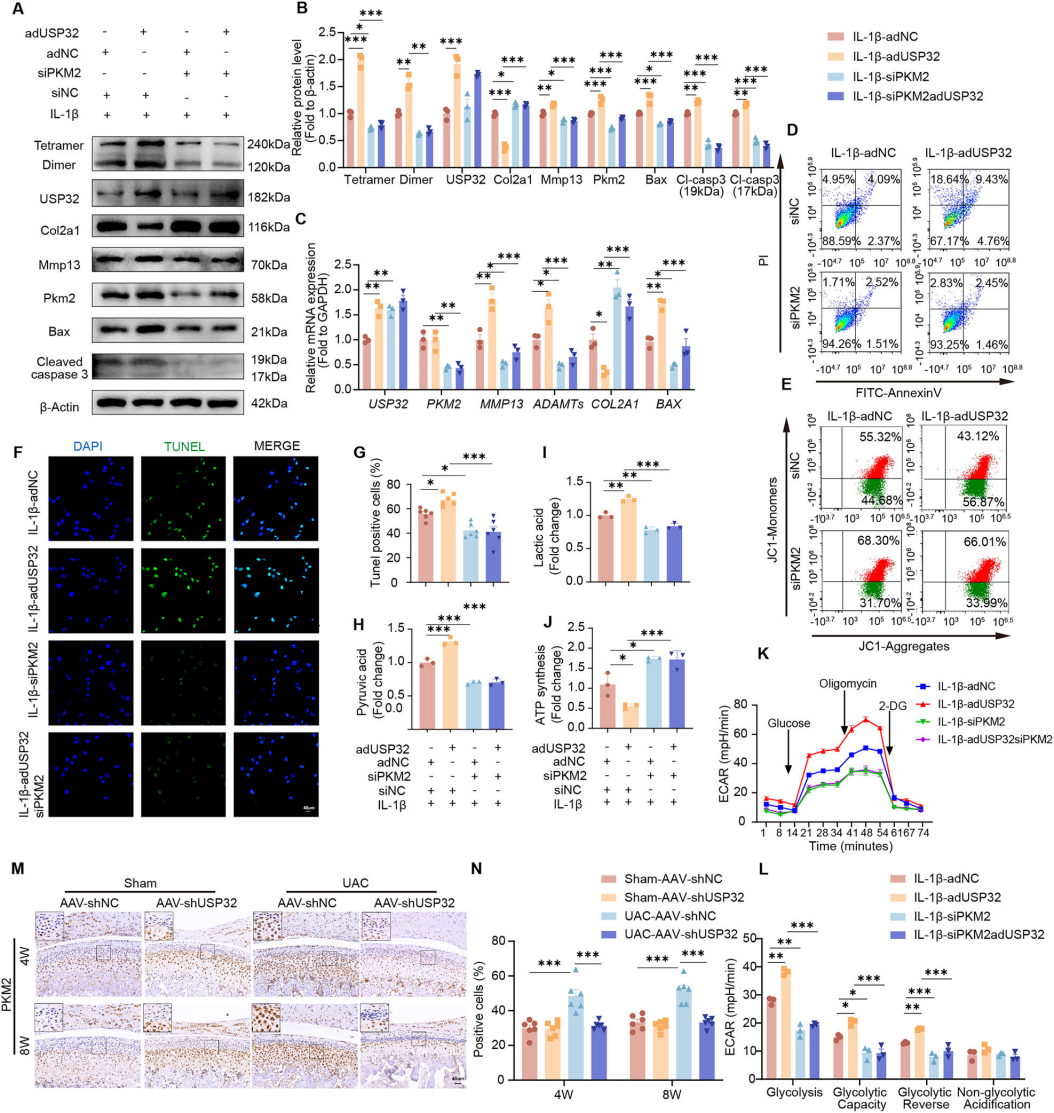
Silencing of PKM2 attenuates the detrimental effects of USP32 overexpression on inflammatory chondrocytes. **A** Representative western blot and quantitative analysis of USP32, PKM2, PKM2 tetramers/dimers, Col2a1, Mmp13, Bax, and Cleaved caspase 3 in inflammatory chondrocytes following transfection with adUSP32 at a multiplicity of infection (MOI) of 150 to overexpress USP32, siPKM2 to silence PKM2, or both. **B** Quantification of protein expression levels (*N* = 3). **C** RT-qPCR analysis of mRNA expression levels of *USP32*, *PKM2*, *MMP13*, *ADAMT*s, *COL2A1*, and *BAX* across experimental groups (*N* = 3). **D** Flow cytometric assay to estimate cellular apoptosis using Annexin V/PI staining in respective groups. **E** Flow cytometry analysis of JC-1 staining to evaluate mitochondrial membrane potential. **F** TUNEL staining illustrating apoptotic cells (green) and nuclei (blue, DAPI), with corresponding quantitative analysis of TUNEL-positive cells (**G**) (*N* = 6). Scale bar 40 μm. **H** Quantification of pyruvate levels in different groups (*N* = 3). **I** Quantification of lactate production across groups (*N* = 3). **J** Measurement of ATP synthesis in experimental groups (*N* = 3). **K** Seahorse extracellular flux analysis depicting extracellular acidification rate. **L** Quantitative assessment of glycolysis, glycolytic capacity, glycolytic reserve, and non-glycolytic acidification rate in respective groups. **M** Immunohistochemical staining for PKM2 in sagittal central sections of the condylar cartilage after delivery of AAV-shUSP32 or AAV-shNC (*N* = 6). Quantitative analysis was shown in (**N**). Scale bar 40 μm. Statistical significance is indicated by * *P* < 0.05, ** *P* < 0.01, *** *P* < 0.001.

To elucidate whether USP32 exerts its inflammatory effects through the regulation of PKM2, we co-transfected siPKM2 in USP32-overexpressing inflammatory chondrocytes (Supplementary Table 5). This strategy enabled us to directly assess the role of PKM2 in USP32-mediated inflammatory responses. Genetic suppression of PKM2 in USP32-overexpressing chondrocytes effectively reversed the ECM degradation, reinstated Col2a1 expression, and suppressed markers like Mmp13, ADAMTs, and apoptotic indicators such as Bax and Cleaved caspase 3 (Fig. 6A–C). This intervention also improved chondrocyte viability and mitochondrial function (Fig. 6D–G Fig. S7A–E) and normalized metabolic perturbations, evidenced by decreased pyruvate and lactate accumulation, as well as restored ATP production (Fig. 6H, J). Furthermore, USP32 specifically enhanced glycolytic activity, as shown by increased ECAR and glycolytic capacity, with these effects being entirely dependent on PKM2, as shown by Seahorse metabolic flux analysis (Fig. 6K, L). In vivo immunohistochemical analysis revealed that PKM2 levels were reduced following the knockdown of USP32 in TMJOA, indirectly validating the impact of USP32 on PKM2 in vivo (Fig. 6M, N).

**Fig. 7:**
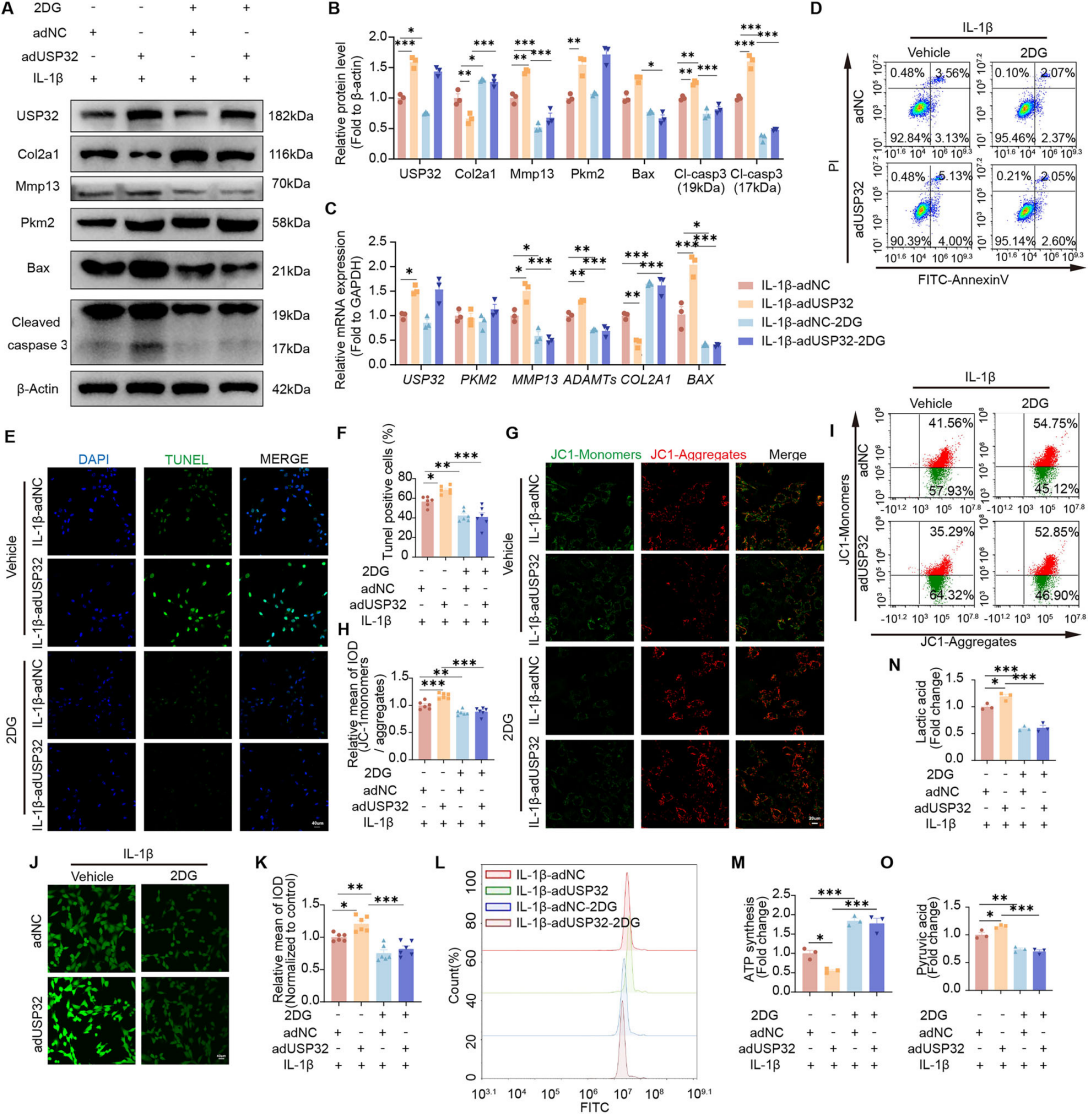
Competitive inhibition of glycolysis by 2-Deoxy-D-Glucose (2-DG) reverses the pathogenic effects of USP32 overexpression in inflammatory chondrocytes. **A** Representative western blot and quantitative analysis of USP32, Col2a1, Mmp13, PKM2, Bax, and Cleaved caspase 3 in inflammatory chondrocytes transfected with adUSP32 to overexpress USP32 or adNC for negative control, followed by treatment with 2-DG (5 mM) or vehicle control. **B** Quantification of protein expression levels (*N* = 3). For Bax protein expression, which showed non-normal distribution, statistical analysis was performed using the Kruskal-Wallis test followed by Dunn’s post hoc correction for multiple comparisons. **C** Quantitative RT-qPCR analysis of mRNA expression levels of *USP32*, *PKM2*, *MMP13*, *ADAMTs*, *COL2A1*, and *BAX* across respective groups (*N* = 3). **D** Flow cytometric evaluation of apoptosis using Annexin V/PI staining. **E** TUNEL staining showing apoptotic cells (green) and nuclei (blue, DAPI) with quantitative analysis of TUNEL-positive cells (*N* = 6) in (**F**). Scale bar 40 μm. **G** JC-1 staining to assess mitochondrial membrane potential, with quantitative analysis (*N* = 6) (**H**). Scale bar 20 μm. **I** Representative flow cytometry assessment of JC-1. **J** Fluorescence intensity changes of ROS levels with quantitative analysis shown in (**K**). Scale bar 40 μm. **L** Flow cytometric measurement of intracellular ROS levels. **M** Measurement of ATP synthesis levels across experimental groups. **N** Quantification of lactate production in different treatment groups. **O** Quantification of pyruvate levels in respective groups. Statistical significance is indicated by *P < 0.05, ** *P* < 0.01, *** *P* < 0.001.

Beyond its involvement in glycolysis, PKM2 may also play a role in influencing gene transcription within inflammatory chondrocytes [27]. To specifically explore the glycolytic regulation of USP32, we employed the competitive glycolysis inhibitor 2-deoxy-D-glucose (2DG), which allowed for a direct comparison of metabolic and transcriptional effects. Inhibition of glycolysis with 2DG significantly diminished the impacts of USP32 overexpression, leading to increased Col2a1 expression while concurrently reducing markers of ECM degradation, such as ADAMTs and MMP13, as well as apoptosis-related proteins (Fig. 7A–C). Simultaneously, treatment with 2DG enhanced chondrocyte viability, as evidenced by a decrease in apoptosis (Fig. 7D–F), a restoration of mitochondrial membrane potential (Fig. 7. G–I), and a reduction in ROS levels (Fig. 7J–L). Additionally, the release of lactate and pyruvate was reduced, while ATP synthesis was enhanced (Fig. 7M–O). These findings establish that USP32-mediated metabolic reprogramming through PKM2 stabilization represents a key mechanism driving inflammatory responses in chondrocytes, and the application of glycolysis inhibitors effectively mitigates its damaging effects on chondrocytes.

## Discussion

**Fig. 8:**
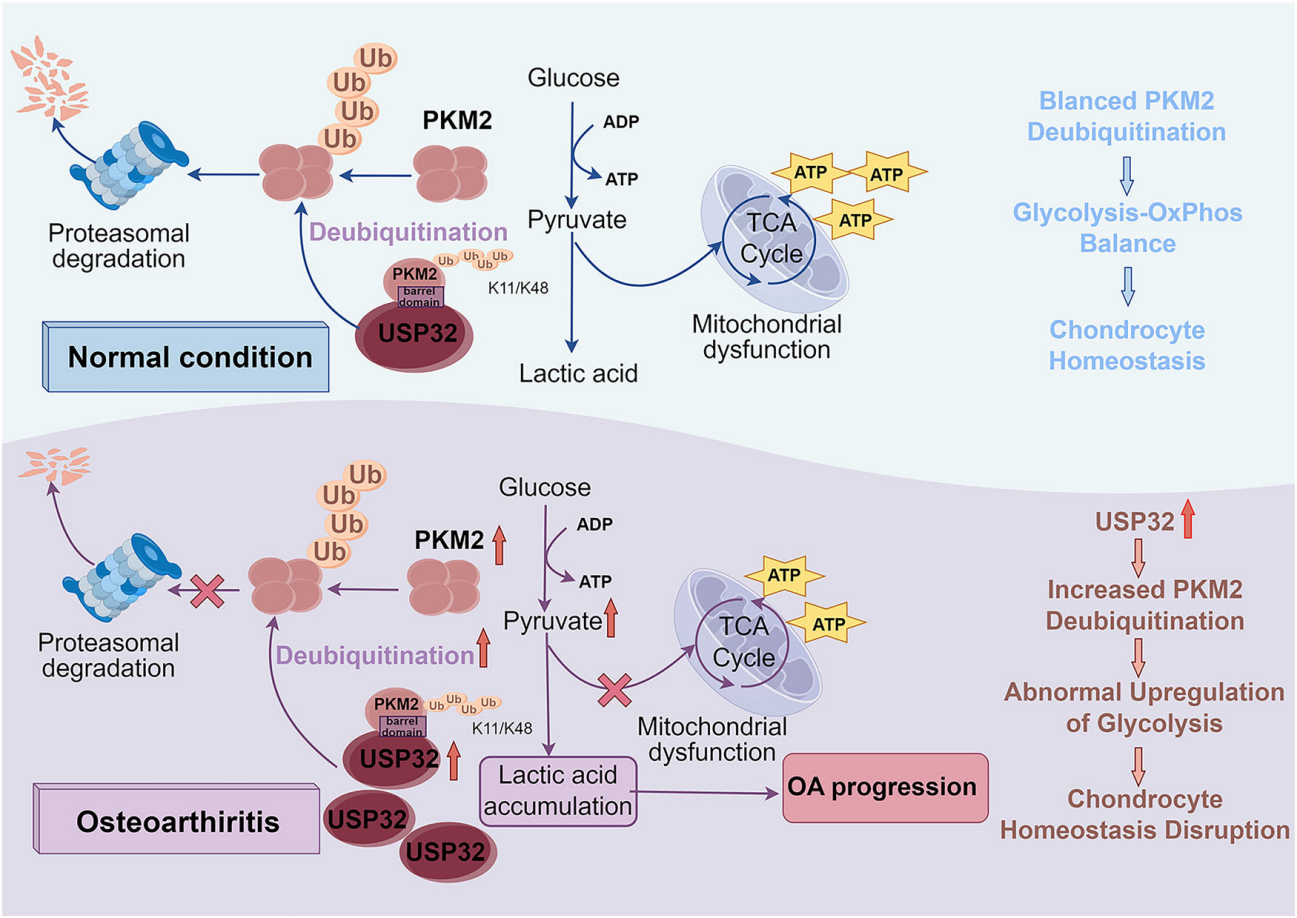
Schematic illustration of the regulatory role of the USP32-PKM2 axis in temporomandibular joint osteoarthritis (TMJOA) pathogenesis. This diagram illustrates the regulatory role of the ubiquitin-specific protease 32 (USP32)-pyruvate kinase M2 (PKM2) axis in TMJOA progression. Under pathological conditions, USP32 is upregulated in TMJOA cartilage and inflammatory chondrocytes, where it stabilizes PKM2 by removing K48- and K11-linked ubiquitin chains, preventing its proteasomal degradation. Excessive accumulation of PKM2 promotes glycolysis and lactate accumulation while impairing mitochondrial function. The metabolic shift exacerbates chondrocyte apoptosis and extracellular matrix (ECM) degradation, contributing to TMJOA pathology.

In this study, emerging evidence suggests that USP32 is the most significantly upregulated DUB in both UAC-induced TMJOA and IL-1β-induced chondrocytes, contributing to enhanced cartilage inflammation, activation of glycolysis, and chondrocyte apoptosis. USP32 specifically targets the barrel domain of PKM2 and selectively cleaves K11- and K48-linked ubiquitin chains, thereby stabilizing PKM2 and enhancing its catalytic activity in glycolytic flux Fig. 8. This novel finding fills a previously unexplored gap in our understanding of USP32’s role in cartilage inflammation and reveals a new metabolic regulatory mechanism in OA pathogenesis. It advances our understanding of the intersection between ubiquitination and metabolic regulation in cartilage homeostasis.

Previous research has established that deubiquitination processes regulate chondrocyte inflammation through mechanisms such as NF-κB activation [39, 40], ER stress induction, NLRP3 modulation [38], and SOX9 regulation [41], ultimately leading to ECM degradation [40–44]. Our study identified USP32 as a previously unrecognized regulator in TMJOA. Although no prior research has been conducted in the context of OA, USP32 has been reported to be associated with cancer progression [45], the regulation of endosomal transport and recycling through its deubiquitylation activity on Rab7 and mTORC1 activation, as well as processes potentially linked to autophagy and inflammation responses [13, 14]. However, these studies have predominantly focused on tumor and immune cells, leaving the function of chondrocytes unexplored. USP32 has been reported to regulate mTORC1 activation and induce autophagy [14], a process that fluctuates dynamically during OA progression [30]. In contrast, our study shows that USP32 is progressively upregulated in correlation with both OA severity and chondrocyte apoptosis. Moreover, the use of glycolysis inhibitors effectively alleviates the pro-inflammatory effects of USP32, highlighting its pathogenic role in metabolic dysregulation. This study is the first to connect USP32 with the regulation of cellular metabolism, providing novel insights into the metabolic influence of USP32 on chondrocytes and its implications in OA.

Metabolic reprogramming in chondrocytes has emerged as a key factor in the progression of OA. The shift from oxidative phosphorylation to glycolysis in chondrocytes, mediated by key enzymes including PKM2 [24], LDHA [46], and PFKFB3 [47], contributes to OA progression through lactate accumulation and reduced ATP production due to the lower efficiency of glycolysis compared to oxidative phosphorylation (OXPHOS) [48]. In this study, mitochondrial dysfunction with reduced ATP and lactate accumulation was observed in OA, while treatment with 2DG attenuated inflammation and apoptosis, consistent with prior findings [24, 34]. However, in certain studies, activation of glycolytic has been shown to confer protective effects and support energy supply during diseases [49, 50]. Yang et al. reported glycolysis inhibition exacerbates OA progression, potentially reflecting age-related factors and model-specific responses [24]. Previous studies have observed that enhancing glycolytic activity contributes to the maintenance of metabolic homeostasis in adult cardiomyocytes [51]. In contrast to cardiac tissues, where impaired glycolysis disrupts the tricarboxylic acid cycle (TCA) [52], chondrocytes, which reside in hypoxic environments, exhibit relatively limited OXPHOS [34]. Hyperactive glycolysis in chondrocytes disrupts metabolic homeostasis, leading to significant intracellular acidification that further inhibits TCA cycle activity [53, 54].

While PKM2 phosphorylation has been extensively studied, its regulation by ubiquitination remains less characterized. We report for the first time that USP32 functions as a DUB, stabilizing PKM2 in both tetramer and dimer forms, as confirmed by LC-MS and immunoprecipitation. Previous studies have demonstrated that FSTL1 regulates PKM2 stability and nuclear translocation via phosphorylation and deubiquitination [55]. In this study, we found that USP32 enhances the stability of PKM2, particularly its tetramerization. The excessive accumulation of tetramers, along with dimer-induced oxidative stress and tetramer-driven glycolysis, may collectively disrupt metabolic homeostasis [56]. Deng et al. have found that excessive dimerization of PKM2, due to the lack of SIRT1, exacerbates OA progression by activating β-catenin-mediated transcriptional programs [27]. In contrast to our research, their study primarily focused on pathological PKM2 dimer accumulation under conditions of stable total PKM2 expression. Notably, our study reveals that PKM2 levels are elevated in OA, and USP32-mediated deubiquitination further promotes the formation of both PKM2 tetramers and dimers. The pathological accumulation of PKM2 dimers and tetramers may disrupt metabolic homeostasis through the combined effects of dimer-mediated redox imbalance and tetramer-driven hyperactive glycolysis. Importantly, our work provides the first evidence of deubiquitination-mediated regulation of PKM2 stability and identifies USP32 as a key metabolic regulator in the pathogenesis of OA.

Although this study reveals USP32’s role in the pathogenesis of TMJOA, several limitations must be considered. The specific interaction domains between USP32 and PKM2 remain uncharacterized, and in vivo rescue experiments were not conducted to fully validate the mechanistic relationship. Additionally, the findings are based on limited in vivo models without validation in human tissue, which may limit their translational relevance. Future studies addressing these aspects will be crucial for strengthening the clinical and mechanistic significance of the USP32-PKM2 axis in TMJOA.

This study identifies USP32 as a key regulator in OA, demonstrating its role in stabilizing PKM2 by removing K48/K11-linked ubiquitin chains and activating glycolysis in chondrocytes. Our findings highlight the USP32-PKM2 axis in the pathogenesis of TMJOA, revealing a novel mechanism of metabolic dysregulation and identifying potential therapeutic targets for OA intervention.

## Supplementary information

Supplementary data

Supplementary Figure 1

Supplementary Figure 2

Supplementary Figure 3

Supplementary Figure 4

Supplementary Figure 5

Supplementary Figure 6

Supplementary Figure 7

Supplementary Figure 8

Supplementary Table 1

Supplementary Table 2

Supplementary Table 3

Supplementary Table 4

Supplementary Table 5

Original Western blots

## Data Availability

The data are available from the corresponding author upon reasonable request.
