# Supplementary material for: USP32 promotes temporomandibular joint osteoarthritis by modulating PKM2 stability and glycolytic metabolism in chondrocytes
Source: Cell Death Dis. 2025 Nov 3;16(1):781. doi: 10.1038/s41419-025-08053-6 (PMC12583448; doi:10.1038/s41419-025-08053-6)
Supplement: Supplementary file 1 — Supplementary data [file 41419_2025_8053_MOESM1_ESM.docx]

***Supplementary data- materials & methods, supplementary figures and tables***

***The supplementary materials primarily include experimental methods (e.g., Western blot, RT-qPCR). Additionally, validation data from in vivo and in vitro experiments are provided, such as the concentration-dependent drug efficacy evaluation, adenovirus-mediated USP32 overexpression and key reagents (e.g., PCR primers, siRNA sequences) shown in the figures and tables.***

**Western blot analysis and Co-IP**

The chondrocytes and cartilage tissue were washed with PBS, smashed, and lysed in RIPA buffer supplemented with protease inhibitor cocktail. Proteins extracted from cells and tissues were then subjected to sodium dodecyl sulfate-polyacrylamide gel electrophoresis (SDS-PAGE). Primary antibodies were used, including anti-USP32(1:100, Santa Cruz, sc-374465), anti-PKM2(1:1000, Proteintech, 15822-1-AP), anti-Col2a1(1:500, Bioss, bs-10589R), anti-Mmp13(1:1000, Proteintech, 18165-1-AP), anti-β-Actin (1:1000, Proteintech, 66009-1-Ig), anti-Bax (1:1000, Proteintech, 50599-2-Ig), anti-Cleaved caspase 3(1:1000, Proteintech, 25128-1-AP), anti-Ub (1:1000, Cell signaling technology, 3936), anti-Eno1(1:1000, ABclonal, A11448), anti-C1qbp(1:1000, ABclonal, A1883), anti-Slc25a4 (1:1000, ABclonal, A15027), anti-Slc25a38(1:1000, ABclonal, A13218), anti-Flag(1:1000, Proteintech, 20543-1-AP), and anti-Myc (1:1000, Proteintech, 60003-2-Ig).

For Co-IP, proteins were extracted from MCCs or HEK-293T cells with 1 mL RIPA buffer. The antibody was treated with 30 μl Protein A+G Magnetic beads (Cat. No: HY-K0202, MedChemExpress, USA) on a vibrator at 4℃ for 12 hours to allow antibody-bead conjugation. Subsequently, 200 μl of protein lysate (1 mg/ml total protein concentration) was added to the antibody-bead complex and incubated on a vibrator at 4°C for an additional 12 hours to facilitate antigen-antibody binding. The IgG antibody (Proteintech, B900620, 98136-1-RR) was employed as a negative control in immunoprecipitation assays to account for nonspecific binding. After incubation, the samples were placed on a magnetic stand (Cat. No: HY-K0200, MedChemExpress, USA) to separate proteins specifically bound to the magnetic beads. Finally, the immunoprecipitated proteins were eluted from the beads using 2×SDS loading buffer by boiling at 95°C for 10 minutes, followed by centrifugation at 12,000 g for 1 minute to collect the supernatant for downstream applications.

**RNA extraction and RT-qPCR**

Total cellular and cartilage tissue RNA was extracted using TRIzol (SparkJade). Following the addition of chloroform, the mixture was vigorously vortexed for 15 seconds to ensure complete homogenization. After incubation at room temperature for 15 minutes, the samples were centrifuged at 12,000 × g for 10 minutes at 4°C to achieve phase separation. The upper aqueous phase containing RNA was carefully transferred to a fresh RNase-free microcentrifuge tube, and an equal volume of isopropanol was added to precipitate the RNA. The mixture was incubated at room temperature for 15 minutes and subsequently centrifuged at 12,000 × g for 10 minutes at 4°C, resulting in RNA pellet formation at the bottom of the tube. The RNA pellet was washed twice with 75% ice-cold ethanol (prepared with DEPC-treated water) and briefly air-dried in a laminar flow hood for 5-10 minutes. The purified RNA was then dissolved in an appropriate volume of DEPC-treated water and quantified using a spectrophotometer. For cDNA synthesis, 1 µg of total RNA was reverse transcribed using the SPARK scriptⅡ1st Strand cDNA Synthesis kit with gDNA eraser (SparkJade) according to the manufacturer’s protocol. Quantitative real-time PCR was performed in a 20 µL reaction volume containing 2×SYBR Green qPCR Mix (SparkJade) on a real-time PCR system. The thermal cycling conditions consisted of an initial denaturation at 95°C for 10 minutes, followed by 40 cycles of 95°C for 15 seconds and 60°C for 1 minute. cDNA was reverse-transcribed and RT-qPCR was performed according to the manufacturer. The following genes, ACAN, MMP13, COL2A1, ADAMTs, IL-1β, USP32, and PKM2, with primers shown in Supplementary Table 5, were expressed as 2-$\Delta\Delta Ct$, mRNA expression was calculated relative to GAPDH.

**Histopathology and immunohistochemical staining**

The serial sections were subjected to histological staining to evaluate structural and proteoglycan alterations in the articular cartilage. For general histological assessment, sections were stained with hematoxylin and eosin (H&E) following standard protocols. To specifically evaluate proteoglycan content, adjacent sections were stained with Safranin O^1^. OARSI scores and cartilage thickness were evaluated based on the previous study^2^. For immunohistochemical analysis, the avidin-biotin-peroxidase complex method was employed. Briefly, tissue sections were deparaffinized in xylene and rehydrated through a graded ethanol series. Endogenous peroxidase activity was quenched by incubation with 3% hydrogen peroxide for 10 minutes at room temperature. Non-specific binding sites were blocked with 5% normal goat serum in phosphate-buffered saline (PBS) for 30 minutes at 37°C. Sections were then incubated overnight at 4°C with primary antibodies diluted in antibody diluent. The following primary antibodies were used for specific antigen detection: anti-PKM2 (1:200, Proteintech, 15822-1-AP), anti-IL1β (1:200, Proteintech, 26048-1-AP)

**Flow cytometry analyses for cell apoptosis, ROS detection, and mitochondrial membrane potential (MMP)**

To investigate cellular apoptosis, reactive oxygen species (ROS) levels, and mitochondrial membrane potential, flow cytometry analysis was performed using specific fluorescent probes and standardized protocols. Annexin V (FITC, Beyotime, C1062) and PI (PerCP, Beyotime, C1062) were used to detect apoptosis and necrosis. ROS levels were measured using the fluorescent probe 2',7'-dichlorodihydrofluorescein diacetate (DCFH-DA) (FITC, Solarbio, CA1410). Mitochondrial membrane potential was assessed using the JC1(FITC, PerCP, Beyotime, C2006). Fluorescence intensity was quantified using a flow cytometer (Agilent NovoCyte, D3080) equipped with appropriate laser excitation and emission filters, and data analysis was performed using specialized flow cytometry software, NovoExpress (Agilent). This comprehensive approach allowed for the simultaneous assessment of multiple cellular parameters associated with cell death and mitochondrial function. For the cartilage MMP analysis, mitochondria were isolated using the tissue mitochondria isolation kit (Beyotime, C3606) and stained using the JC1(FITC, PerCP, Beyotime, C2006). A microplate reader (BioTek CYTATION-5) was used for quantitative analysis.

**ATP, lactic, and pyruvic acid assessment**

Primary chondrocytes were seeded into 6-well plates and cultured following the specified experimental protocol. Subsequently, the culture medium was aspirated, and cellular ATP levels and pyruvic acid concentrations were quantified utilizing a micro pyruvate acid assay kit (Abbkine, KTB1121) and a chemiluminescence-based ATP assay kit (Beyotime, S0026), respectively. For the analysis of cartilage tissue, samples were homogenized on ice before ATP quantification and pyruvate acid analysis, following the manufacturer’s instructions. Similarly, for lactic acid analysis, primary chondrocytes underwent identical treatment. The supernatant media were collected and centrifuged to eliminate cell debris and suspended cells, after which the secreted lactate levels in the media were measured using a lactic acid assay kit (Elabscience, E-BC-K044-M), following the manufacturer’s protocol. The assays were conducted in 96-well plates, and luminescence signals were detected using a multimode microplate reader (BioTek CYTATION-5). Data acquisition and analysis were performed using Gen5 microplate software (version 3.04, BioTek Instruments).

**Immunofluorescence**

Cells were seeded into confocal plates (Biosharp, BS-20-GJM) and subjected to the previously described experimental protocol. Following this, the cells were washed with PBS and fixed with 4% paraformaldehyde (PFA) for 20 minutes at room temperature and subsequently permeabilized using 0.2% Triton™ X-100 for 15 minutes. The cells were then incubated overnight at 4°C with primary antibodies targeting USP32 (1:200, Santa Cruz, sc-374465) and PKM2 (1:200, Proteintech, 15822-1-AP). After washing with PBS, the cells were incubated with CY3-conjugated secondary antibodies (1:200, BOSTER, BA1031) and FITC-conjugated secondary antibodies (1:200, BOSTER, BA1105) at 37°C for 1 hour. Finally, the cell nuclei were counterstained with DAPI (BOSTER, AR1176) for visualization purposes. Microscopic images were acquired using a laser scanning confocal microscope (Olympus FV3000). Fluorescence intensity colocalization analysis was conducted using ImageJ to quantify the relative fluorescence intensities of both channels within identical regions of interest (ROIs)^3^.

**MicroCT analysis**

Temporomandibular joints from different animals were isolated, fixed overnight in 10% neutral buffered formalin, and washed with phosphate buffer saline (PBS). Joints were then scanned by microCT (Quantum GX), followed by three-dimensional reconstruction with bone volume fraction (BV/TV), trabecular number (Tb.N), which were analyzed, followed by a previous study^4^.

**Transmission electron microscopy**

Transmission electron microscopy (TEM) of joint cartilage was performed to assess ultrastructural morphology, especially the mitochondria. Freshly dissected articular cartilage of sham, UAC group transfected with AAV-shUSP32 was trimmed into pieces around 1mm^3^ and fixed in 2.5% glutaraldehyde (24-48 h, 4°C), post-fixed in 1% osmium tetroxide (1-2 h), and dehydrated in an ethanol series. Tissues were transitioned via propylene oxide, embedded in epoxy resin (Epon 812), and polymerized (60°C, 48 h). Ultrathin sections around 70–90 nm were cut using a diamond knife, mounted on copper grids, and stained with uranyl acetate and lead citrate. Imaging was conducted at 120 kV using a transmission electron microscope (Hitachi, H7650, Tokyo, Japan), with magnifications ranging from 3000× to 5000×.

**Animal selection criteria**

Inclusion criteria required animals to: (1) exhibit normal weight range for age, (2) display no pre-existing orofacial abnormalities upon veterinary examination, and (3) demonstrate normal feeding behavior during a 3-day acclimation period. Exclusion criteria included: (1) presence of spontaneous TMJ dysfunction, (2) failure to regain consciousness within 30 minutes post-anesthesia, or (3) development of surgical complications, for example, wound infection and weight loss >15%. All samples were coded numerically to ensure objective analysis, with group allocation concealed during both experimental procedures and outcome assessments.

**References:**

1. Yang H, Wen Y, Zhang M, Liu Q, Zhang H, Zhang J*, et al.* MTORC1 coordinates the autophagy and apoptosis signaling in articular chondrocytes in osteoarthritic temporomandibular joints. *Autophagy* 2020, **16**(2)**:** 271-288.

2. Zhang M, Wang H, Zhang J, Zhang H, Yang H, Wan X*, et al.* Unilateral anterior crossbite induces aberrant mineral deposition in degenerative temporomandibular cartilage in rats. *Osteoarthritis Cartilage* 2016, **24**(5)**:** 921-931.

3. Schneider CA, Rasband WS, Eliceiri KW. NIH Image to ImageJ: 25 years of image analysis. *Nat Methods* 2012, **9**(7)**:** 671-675.

4. Zhang J, Liao L, Zhu J, Wan X, Xie M, Zhang H*, et al.* Osteochondral Interface Stiffening in Mandibular Condylar Osteoarthritis. *J Dent Res* 2018, **97**(5)**:** 563-570.

**Figure legends of the supplementary figures**

**FigS.1 TMJOA is characterized by cartilage degradation and mitochondrial impairment. A** Schematic representation of the experimental model of unilateral anterior crossbite (UAC)-induced temporomandibular joint osteoarthritis (TMJOA). **B** Representative three-dimensional reconstructions and sagittal central two-dimensional images of joint tissues from sham and UAC groups at 4 and 8 weeks as visualized by micro-computed tomography (Micro-CT). Scale bar 500μm. **C, D** Quantitative morphometric analysis of subchondral bone microstructure, including bone volume fraction (BV/TV), trabecular number (Tb.N) derived from Micro-CT imaging. **E** Quantitative analysis of OARSI score evaluated from HE staining of histological results**. F-I** RNA expression levels of *ACAN*, *COL2A1*, *MMP13*, and *IL-1β* measured by RT-qPCR. **J** Assessment of mitochondrial membrane potential in isolated mitochondria from cartilage using JC-1 staining. Red fluorescence indicates polarized mitochondria, while green fluorescence reflects depolarized mitochondria. Scale bar 200μm. **K** Quantitative analysis of the red(aggregates)/green(monomers) fluorescence ratio using a microplate reader, representing mitochondrial membrane potential integrity. Statistical significance is indicated by **P* < 0.05, ***P* < 0.01, ****P* < 0.001.

**FigS.2** **RNA sequencing in cartilage samples derived from sham and UAC models A** Heatmap of correlation coefficients among samples. **B** Volcano plot of differentially expressed genes (DEGs) from RNA sequencing between sham and UAC groups, red represents upregulation and blue represents downregulation. **C** Bar graph illustrating the number and distribution of DEGs. **D** Hierarchical clustering heatmap of gene expression patterns across samples. **E** Gene Set Enrichment Analysis (GSEA) indicates pronounced enrichment of gene sets associated with carbon metabolism (GO:01200), pyruvate metabolism (GO:00620) in the UAC group. **F** GSEA plots of gene sets related to inflammatory response (GO: 0006954) and regulation of inflammatory response (GO:0050727) enriched in the UAC group. **G** GSEA plots of gene sets related to protein deubiquitination (GO:0016579), and K48(GO:0071108), K63(GO:0071108) linked deubiquitination enriched in the UAC group. **H** GSEA plots of gene sets related to protein ubiquitination (GO:0016567) and K48(GO:0070936), K63(GO:0070534) linked ubiquitination enriched in the UAC group.

**FigS.3** **Chondrocyte-specific knockdown of USP32 reversed cartilage damage in the TMJOA model induced by the UAC A** Schematic illustration of intra-articular injection with trypan blue to represent the injection site of AAV-shNC and AAV-shUSP32 to transfect chondrocytes. **B** Rats were injected with 30 μL of pAV-COL2A1-GFP-mir30-shUSP32 (10E12 vg/ml) into each side of the TMJ at 4 weeks before sample collection. GFP expression indicates successful transduction of chondrocytes. Scale bar 200 μm. **C** Schematic diagram of the adeno-associated virus (AAV) vector applied. **D** Histological analysis of TMJ cartilage at 8 weeks from the Sham and UAC groups after bilateral intra-articular injection of AAV-shNC or AAV-shUSP32. HE staining, Safranin O, and immunohistochemical staining for IL-1β are shown. Statistical results are presented in Fig. 2. Scale bar 40 μm. **E** Statistical measurements of pyruvate levels in cartilage at 4 and 8 weeks from each group. **F** Transmission Electron Microscope (TEM) images of TMJ cartilage at 8 weeks from the Sham, UAC, and UAC + AAV-shUSP32 groups, which demonstrate segmented mitochondria in TMJOA and a recovery of morphology after knockdown of USP32. The below image (magnification, 5000×) shows a magnified view of the structure within the white box above (magnification, 3000×). Statistical significance is indicated by **p < 0.01, ***p < 0.001.

**FigS.4** **Inflammatory chondrocytes exhibited mitochondrial dysfunction and increased apoptosis. A** Protein expression levels of Col2a1, Mmp13, Bax, and Cleaved caspase 3 in primary mandibular condylar chondrocytes (MCCs) after 24-hour treatment with different concentrations of IL-1β. **B** Quantification of protein expression by Western blot analysis (N = 3). **C** RNA expression of *COL2A1*, *MMP13*, and *ACAN* in MCCs treated with different concentrations of IL-1β, measured by RT-qPCR (N = 3) **D** Flow cytometry analysis using Annexin V/PI fluorescence staining of MCCs under different treatment conditions. **E, F** Flow cytometry analysis of ROS levels in MCCs and ATDC5 cells treated with 10 ng/ml IL-1β or a negative control of PBS for 24 hours compared to the control group. **G** Representative fluorescence images of ROS staining in MCC, knee cartilage chondrocytes (KCCs), and ATDC5 cells following inflammatory stimulation with corresponding quantitative analysis shown on the right **(H)**. Scale bar 10 μm. **I** Flow cytometry analysis of JC-1 staining in MCCs under different treatment groups. **J-L** JC-1 fluorescence staining in three kinds of cells treated with IL-1β or negative control, with positive control CCCP treatment, and quantitative analysis results are shown in **(M)**. Scale bar 20 μm. **N** Comparison of culture medium containing phenol red between the inflammation treatment group and control group to compare the lactate acid. Statistical significance is indicated by **P* < 0.05, ***P* < 0.01, ****P* < 0.001.

**FigS.5 Changes in chondrocytes after USP32 overexpression. A** Transfection of MCCs with adenovirus adNC and adUSP32 at a multiplicity of infection (MOI) of 150. Protein expression of USP32, Col2a1, Mmp13, Bax, and Cleaved caspase 3 in different groups, with quantification in panel **(B)** (N = 3). **C** Quantification of RNA expression levels of *USP32*, *MMP13*, *ADAMTs*, *COL2A1*, and *BAX* (N = 3). **D** Annexin V/PI flow cytometry analysis in each group to analyze the apoptosis. **E** TUNEL staining in respective groups, with TUNEL (green) and DAPI (blue) staining, and quantitative analysis on the right **(F)** (N = 6). Scale bar 40 μm. **G** JC-1 mitochondrial membrane potential staining with quantification in the **(H)** (N = 6) and flow cytometry results in panel **(I)**. Scale bar 20 μm. **J** ROS flow cytometry and fluorescence detection **(K)** with quantitative analysis in panel **(L).** Scale bar 40 μm. **M** Lactate assay and ATP quantification **(N)** in different treatment groups. Statistical significance is indicated by **P* < 0.05, **P < 0.01, ***P < 0.001.

**FigS.6 Interaction between USP32 and PKM2. A** Coomassie Brilliant Blue staining of protein gels following co-immunoprecipitation assays using anti-USP32 antibody, with IgG antibody serving as a negative control. **B** Subcellular colocalization of USP32 (red) and PKM2 (green) visualized by inverted fluorescence microscopy, demonstrating their spatial relationship. Scale bar 500μm.  **C** Accuracy assessment of the structural prediction of the USP32-PKM2 interaction using AlphaFold.

**FigS.7 Silencing of PKM2 attenuates the mitochondrial dysfunction of USP32 overexpression in inflammatory chondrocytes. A** Measurement of intracellular ROS levels, accompanied by quantitative analysis in **(B)** (N=6) and flow cytometry histograms **(C).** Scale bar 40μm. **D** JC-1 staining to evaluate mitochondrial membrane potential, accompanied by quantitative analysis in **(E)** (N=6). Scale bar 20 μm. Statistical significance is indicated by ***P* < 0.01, ****P* < 0.001.

**FigS.8 Validation and sequencing of gene silencing. A** Bright-field and fluorescence images of MCCs after 24 hours of transfection with FAM-labeled negative control (FAM-NC). Scale bar 50 μm. **B** Western blot analysis of USP32 protein expression following knockdown with different siRNA sequences, accompanied by RT-qPCR validation of RNA expression levels in **(C)**. **D** Western blot analysis of PKM2 protein expression following knockdown with different siRNA sequences, accompanied by RT-qPCR validation of RNA expression levels in **(E)**. Statistical significance is indicated by ***P* < 0.01, ****P* < 0.001.
