# Supplementary figures and images for: USP32 promotes temporomandibular joint osteoarthritis by modulating PKM2 stability and glycolytic metabolism in chondrocytes
Source: Cell Death Dis. 2025 Nov 3;16(1):781. doi: 10.1038/s41419-025-08053-6 (PMC12583448; doi:10.1038/s41419-025-08053-6)

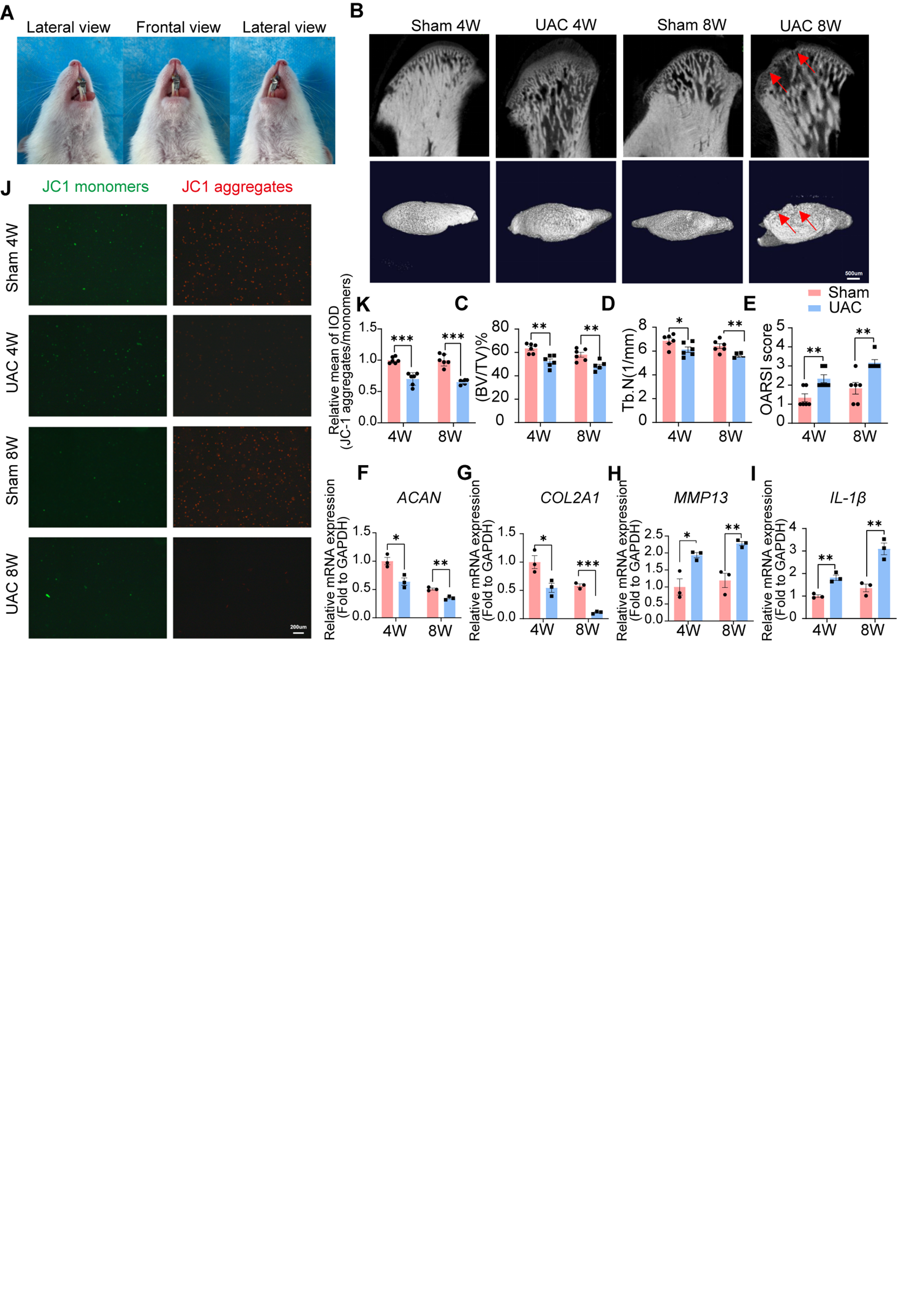

Supplement: Supplementary file 2 — Supplementary Figure 1 [file 41419_2025_8053_MOESM2_ESM.png]

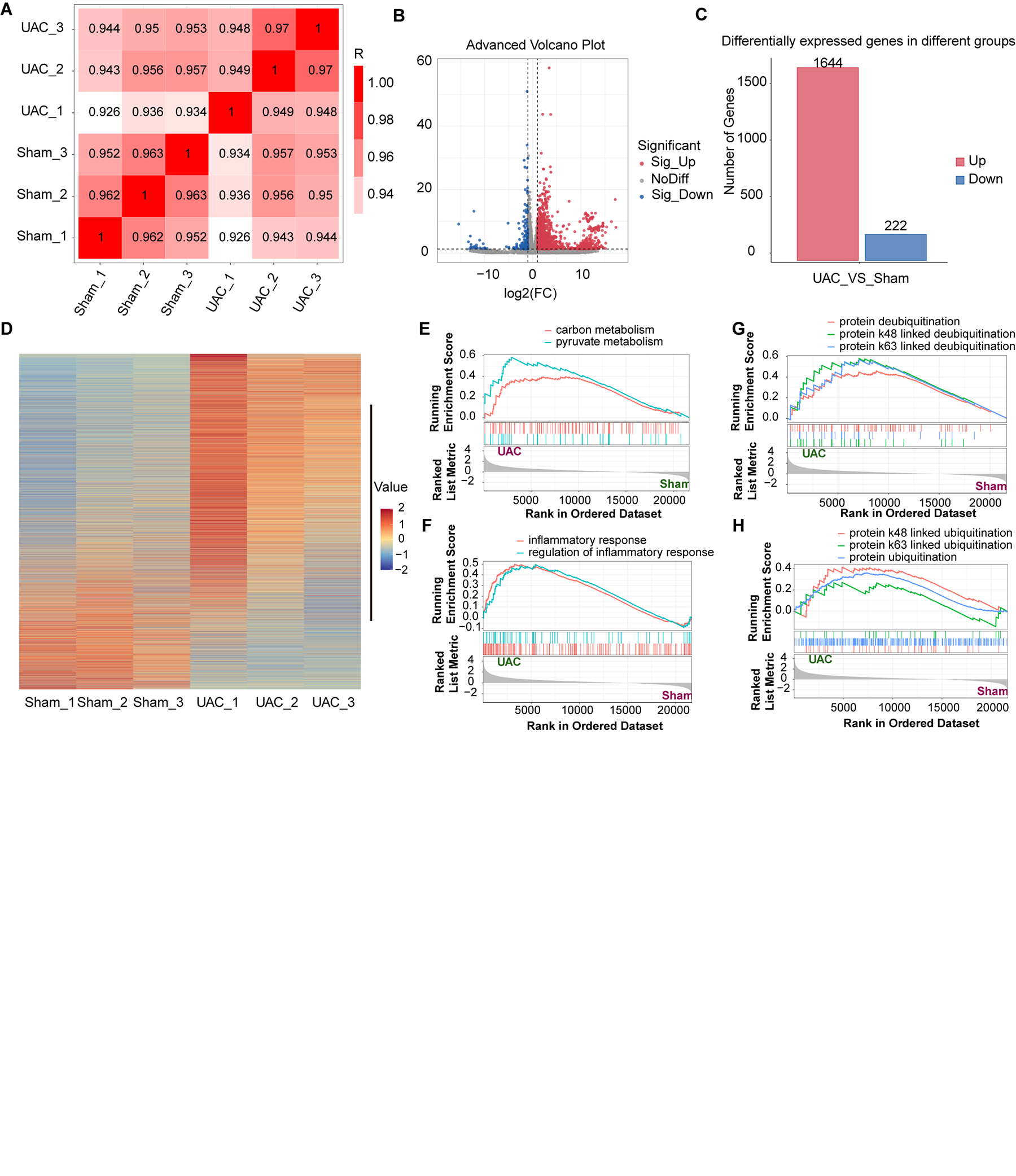

Supplement: Supplementary file 3 — Supplementary Figure 2 [file 41419_2025_8053_MOESM3_ESM.png]

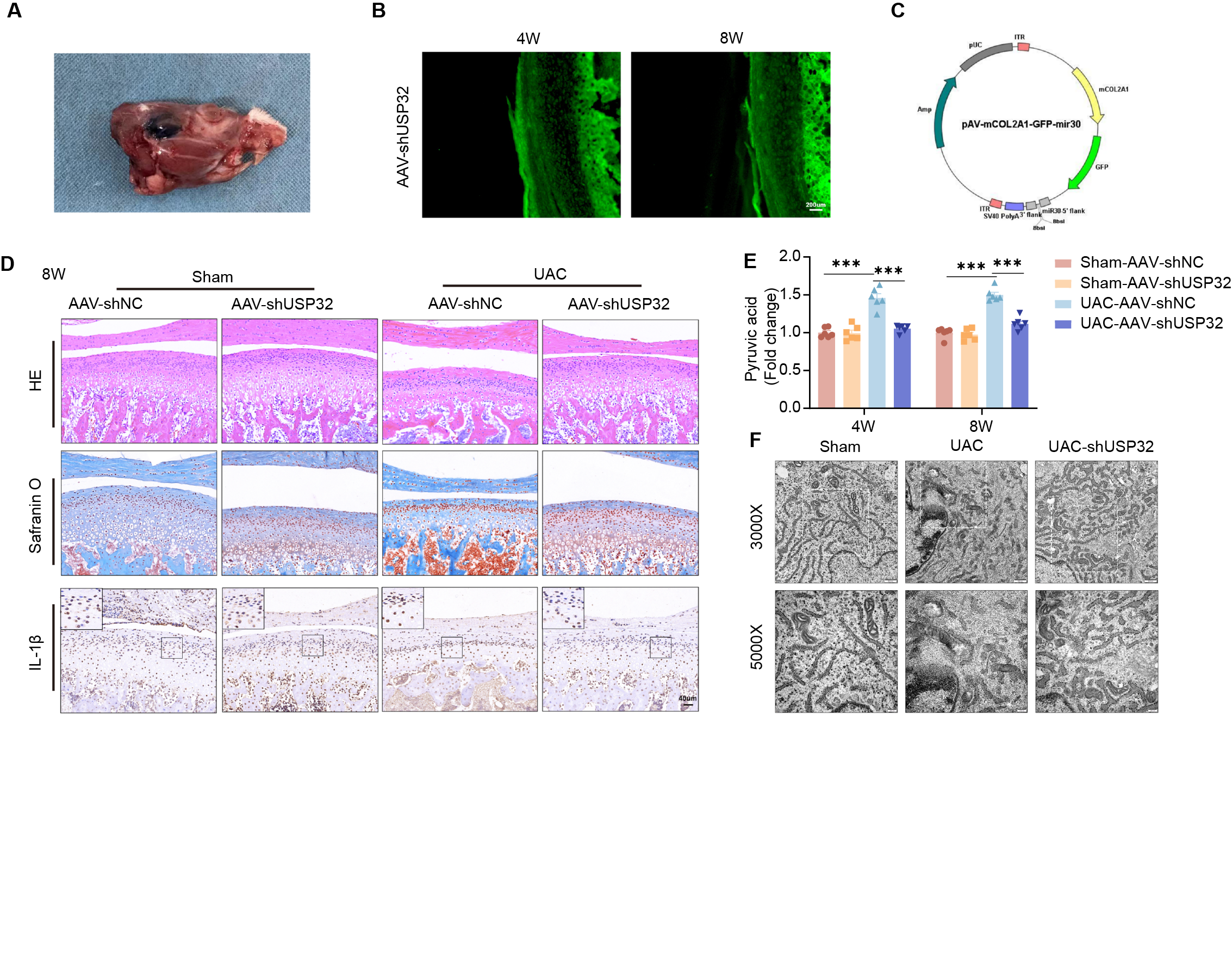

Supplement: Supplementary file 4 — Supplementary Figure 3 [file 41419_2025_8053_MOESM4_ESM.png]

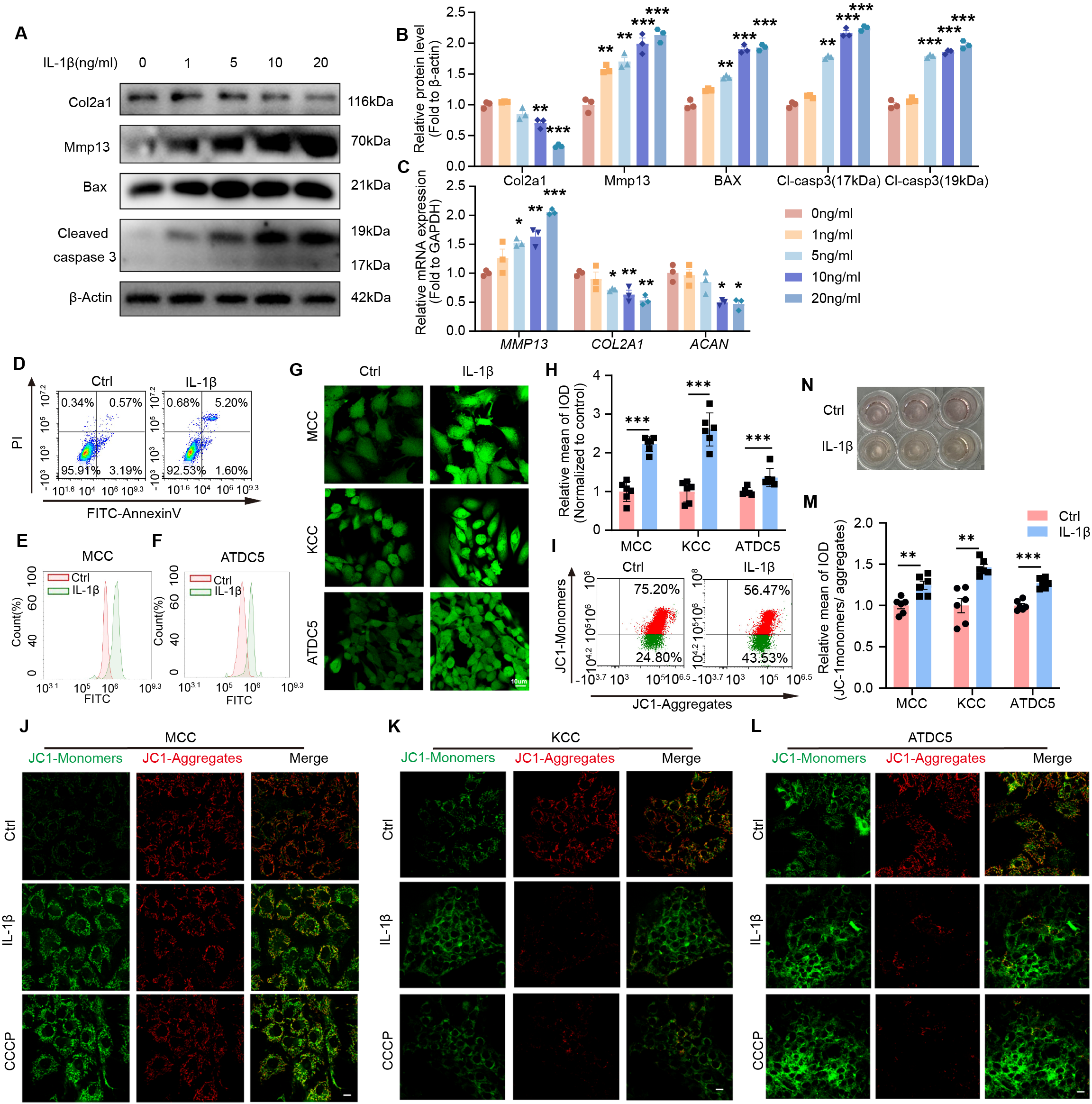

Supplement: Supplementary file 5 — Supplementary Figure 4 [file 41419_2025_8053_MOESM5_ESM.png]

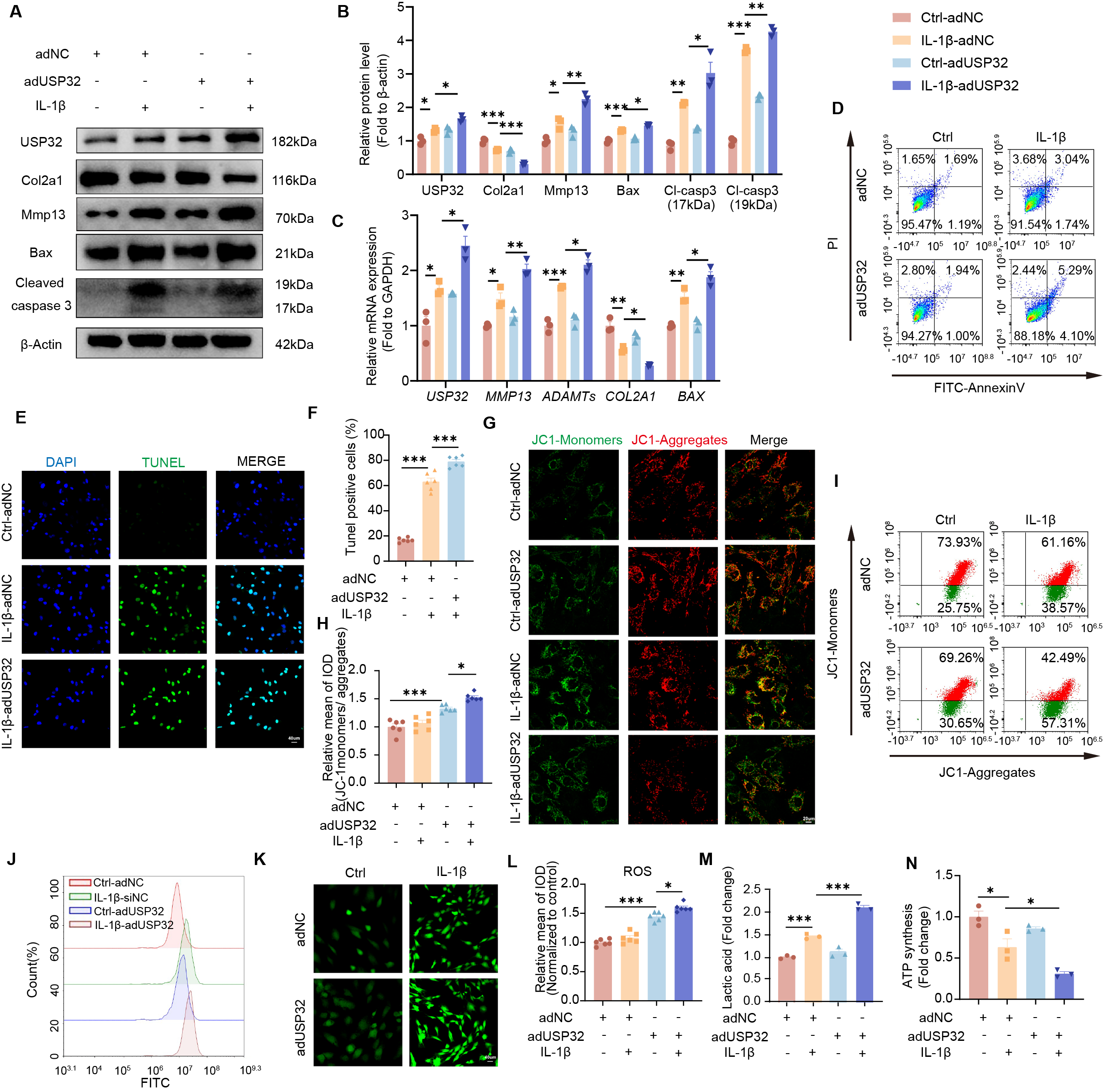

Supplement: Supplementary file 6 — Supplementary Figure 5 [file 41419_2025_8053_MOESM6_ESM.png]

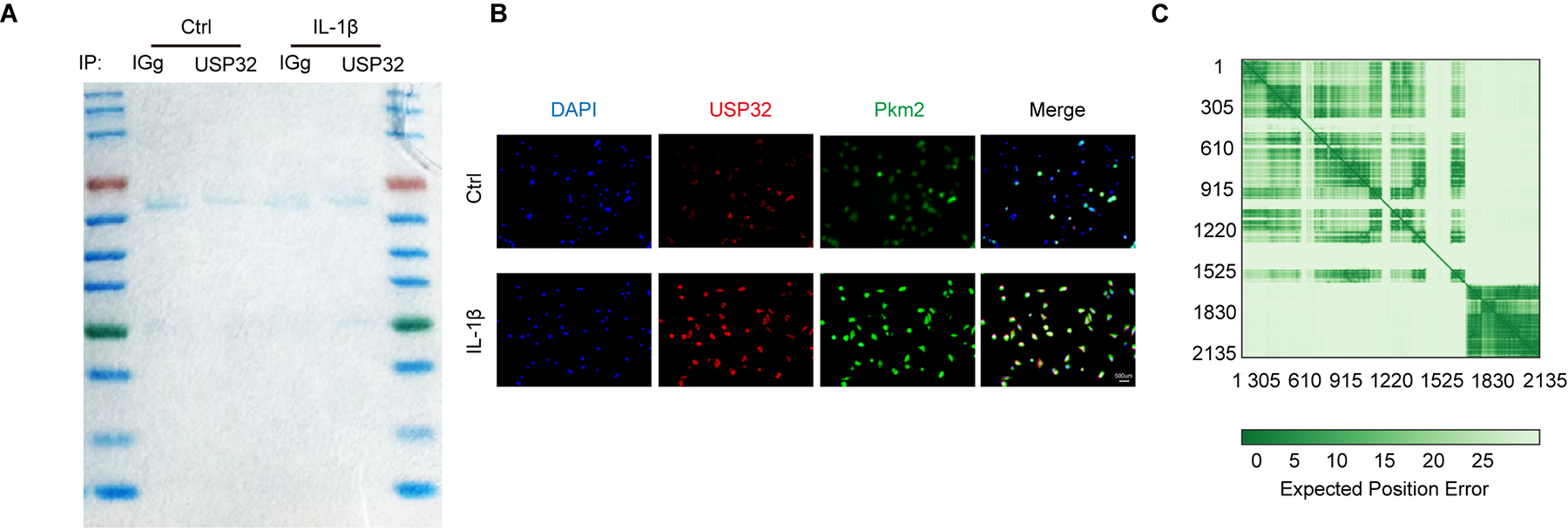

Supplement: Supplementary file 7 — Supplementary Figure 6 [file 41419_2025_8053_MOESM7_ESM.png]

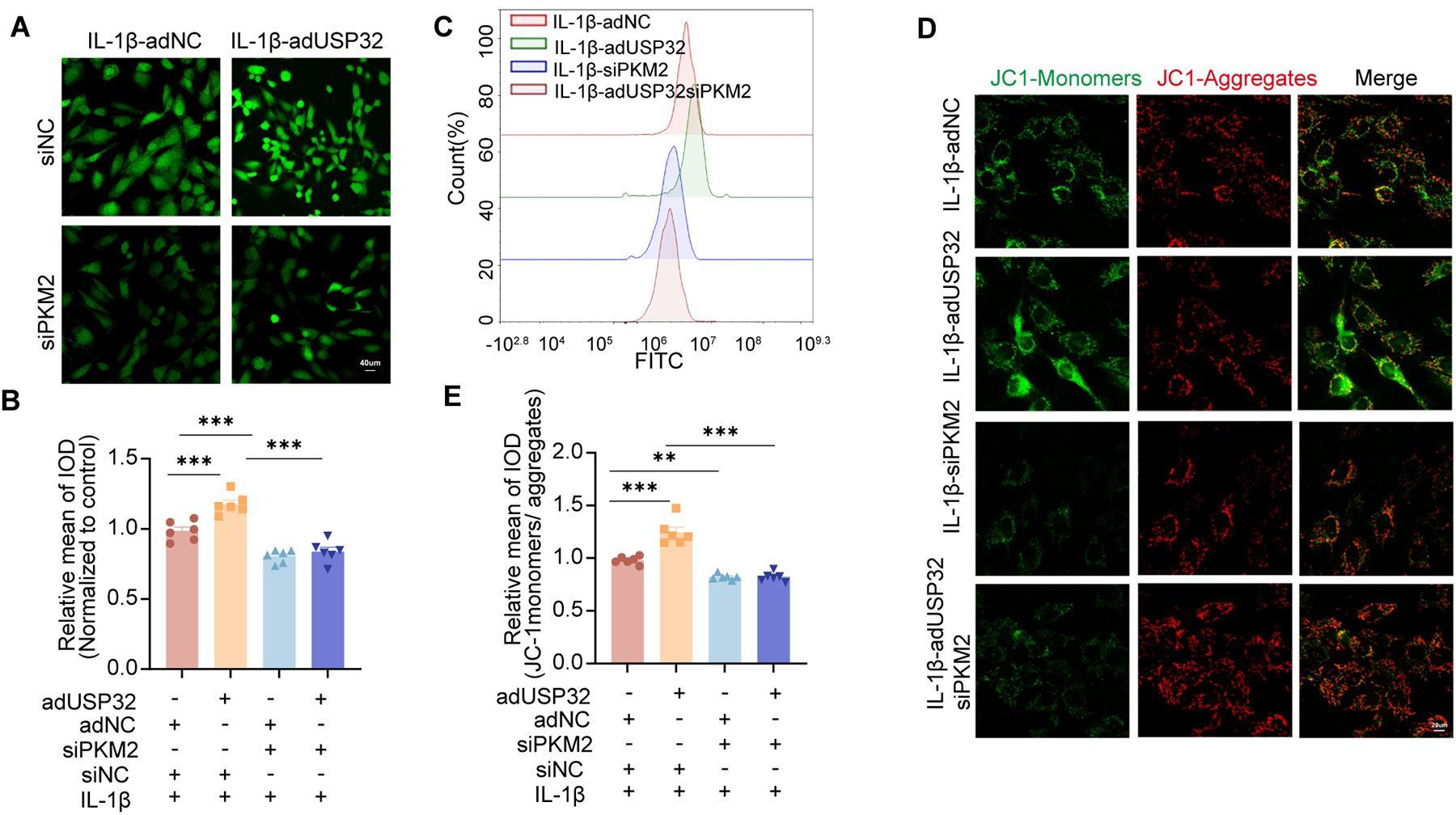

Supplement: Supplementary file 8 — Supplementary Figure 7 [file 41419_2025_8053_MOESM8_ESM.png]

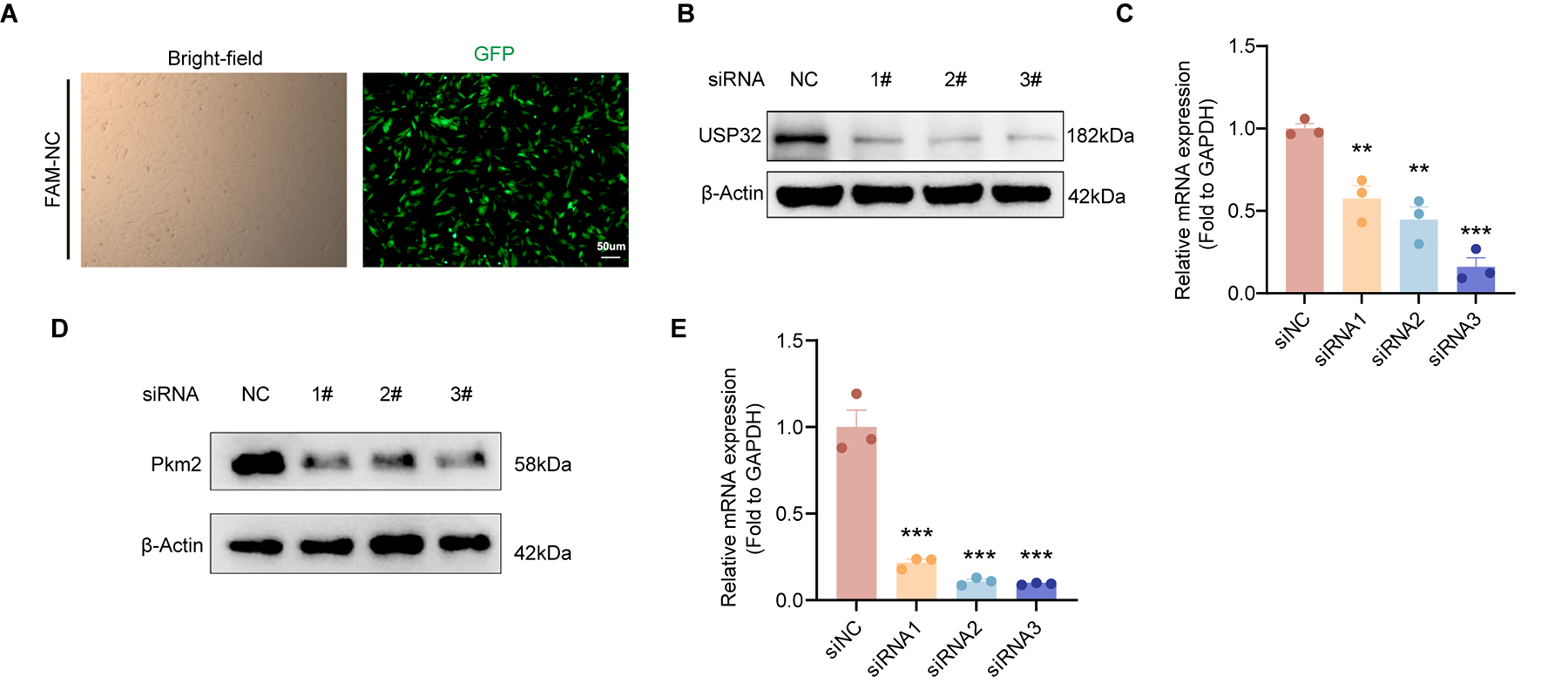

Supplement: Supplementary file 9 — Supplementary Figure 8 [file 41419_2025_8053_MOESM9_ESM.png]
