# Supplementary material for: USP32 promotes temporomandibular joint osteoarthritis by modulating PKM2 stability and glycolytic metabolism in chondrocytes
Source: Cell Death Dis. 2025 Nov 3;16(1):781. doi: 10.1038/s41419-025-08053-6 (PMC12583448; doi:10.1038/s41419-025-08053-6)

Markers (Epizyme #WJ103) was used in WB detection.

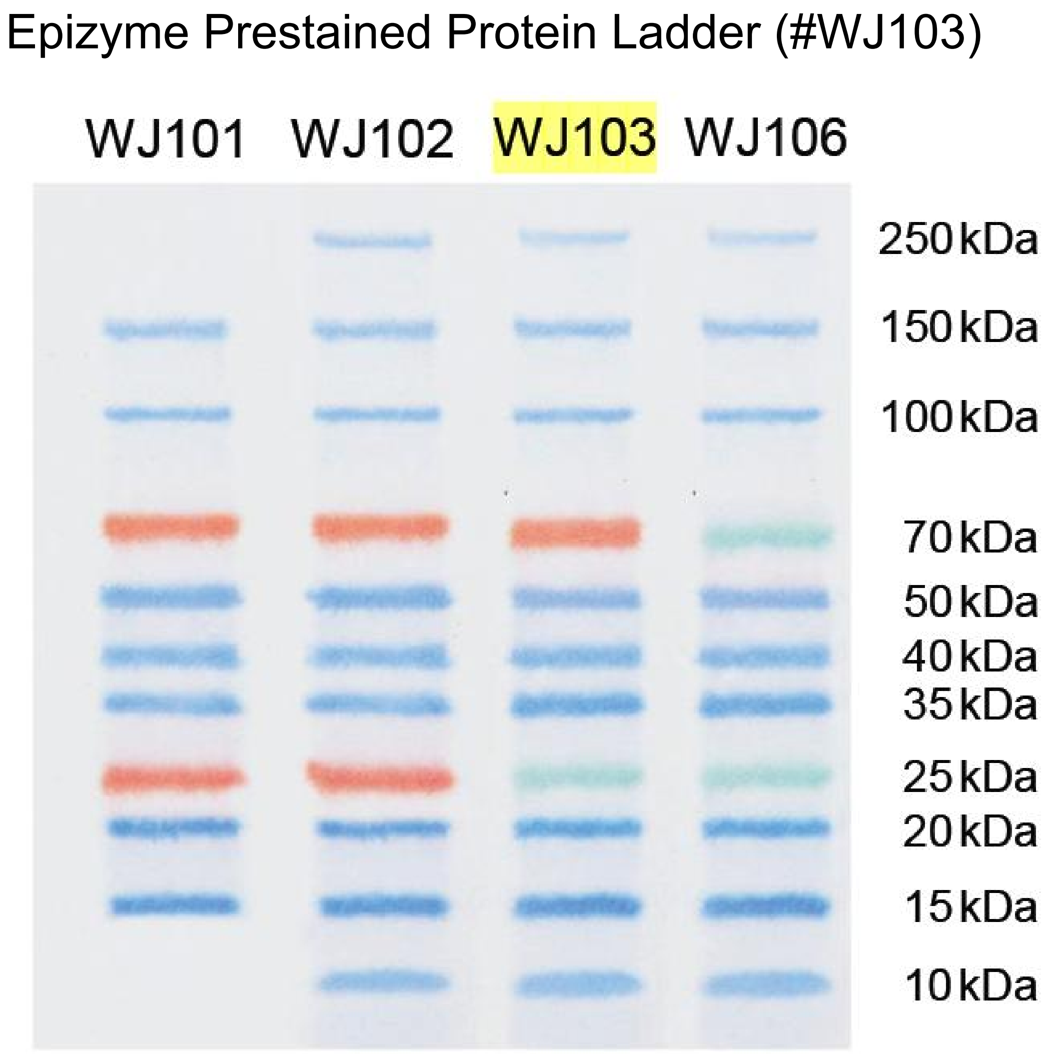

Figure 1F

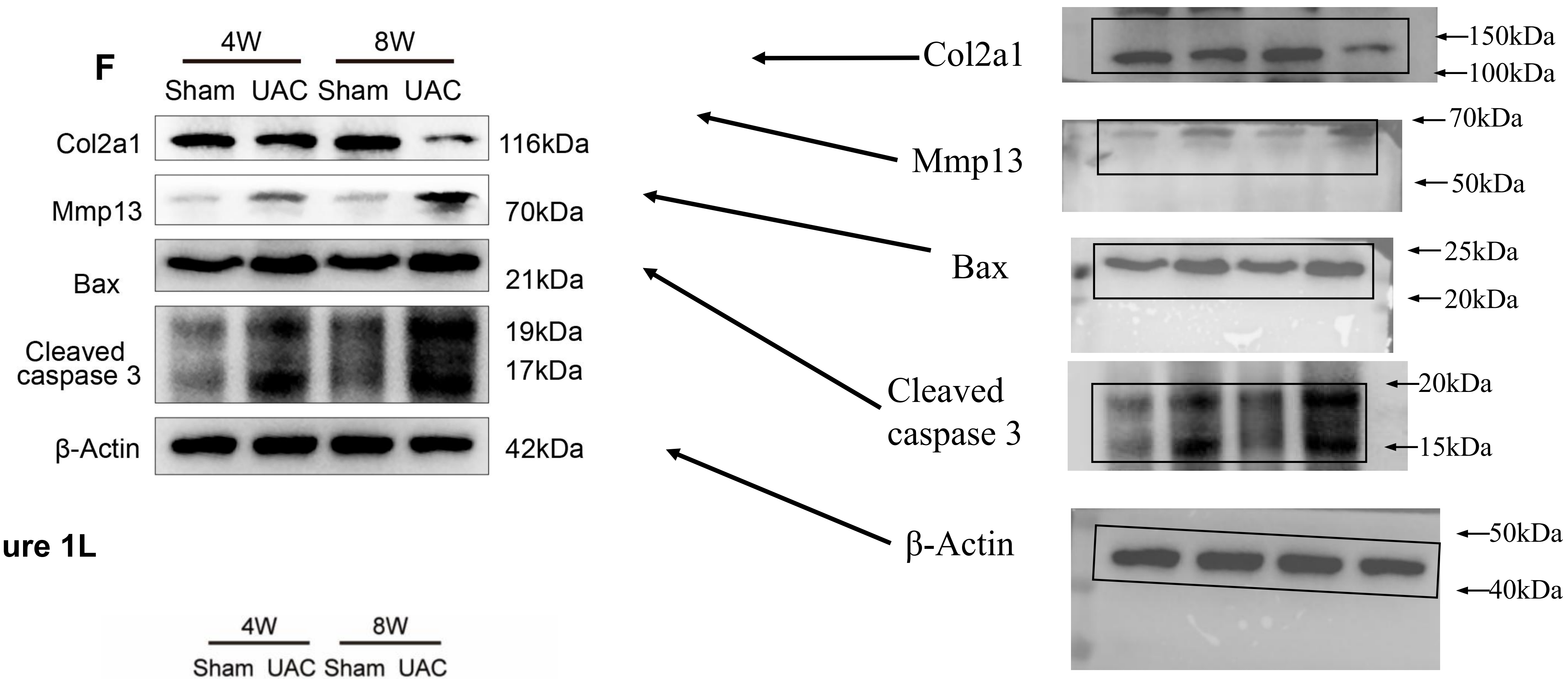

Figure 1L

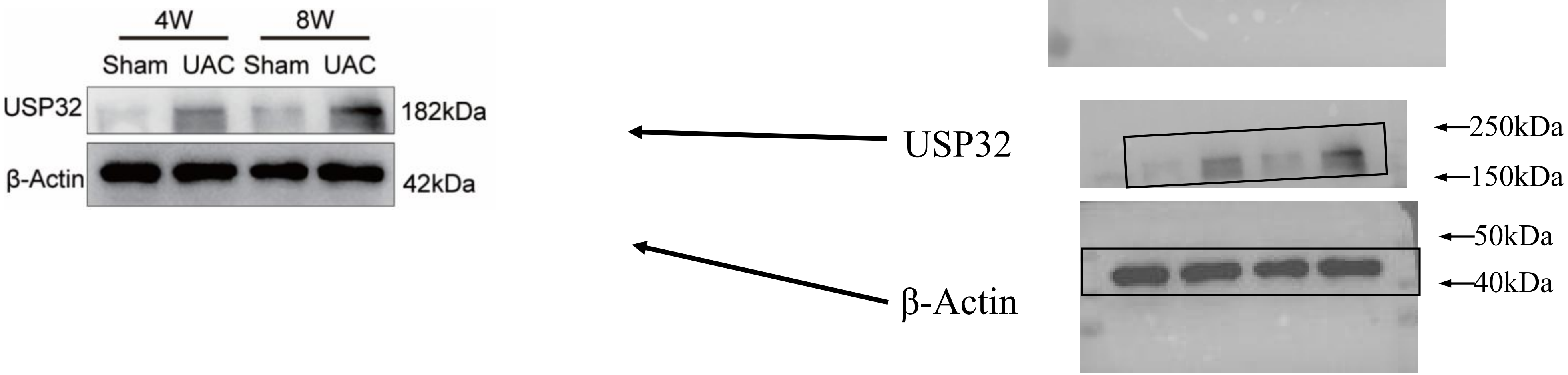

Figure 2A

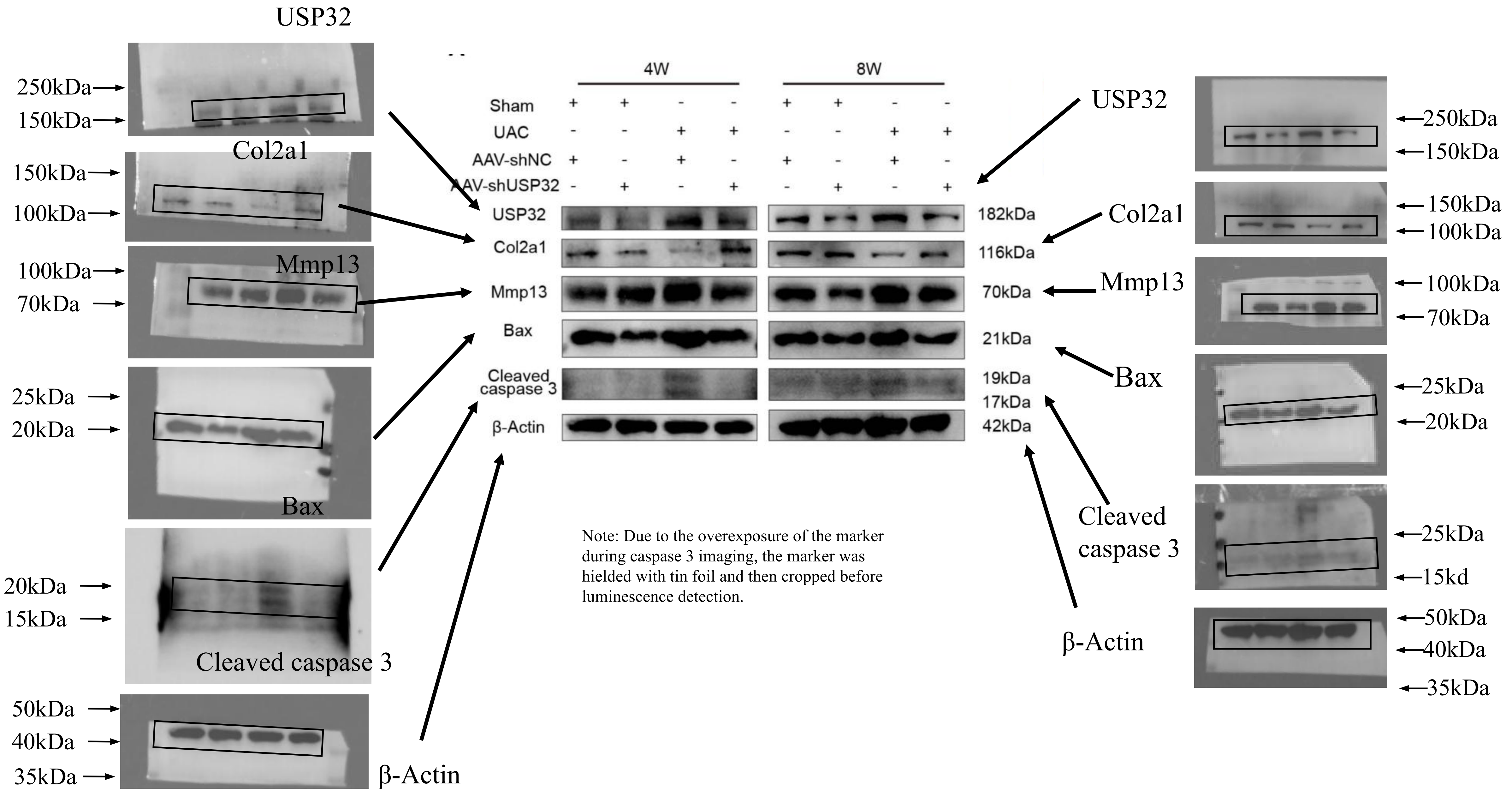
USP32
250kDa
150kDa
Col2a1
150kDa
100kDa
Mmp13
100kDa
70kDa
25kDa
20kDa
Bax
20kDa
15kDa
Cleaved caspase 3
50kDa
40kDa
35kDa
β-Actin
4W
8W
Sham
UAC
AAV-shNC
AAV-shUSP32
USP32
Col2a1
Mmp13
Bax
Cleaved caspase 3
β-Actin
182kDa
116kDa
70kDa
21kDa
19kDa
17kDa
42kDa
Note: Due to the overexposure of the marker during caspase 3 imaging, the marker was hielded with tin foil and then cropped before luminescence detection.
USP32
250kDa
150kDa
Col2a1
150kDa
100kDa
Mmp13
100kDa
70kDa
Bax
25kDa
20kDa
Cleaved caspase 3
25kDa
15kd
β-Actin
50kDa
40kDa
35kDa

Figure 3A

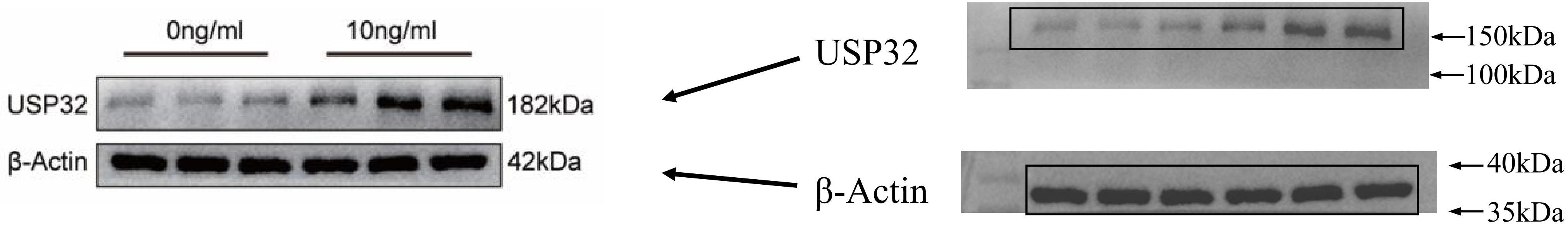

Figure 3D

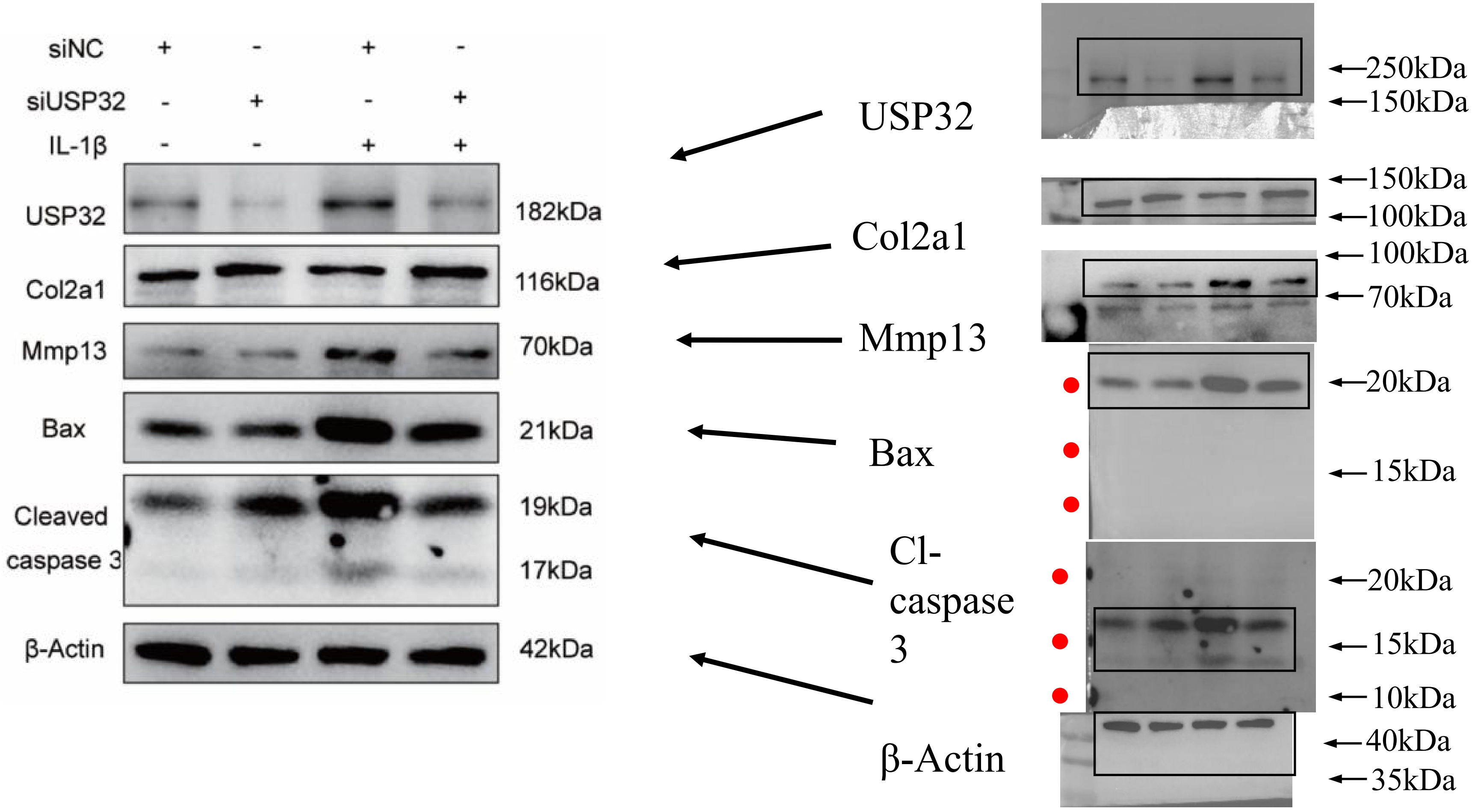

Note: Due to the overexposure of the marker during caspase 3 imaging, the marker was cropped before luminescence detection. As a result, the stripped blot for Bax protein lacks a marker. Please refer to the caspase 3 marker as indicated by the red dot.

Figure 4B

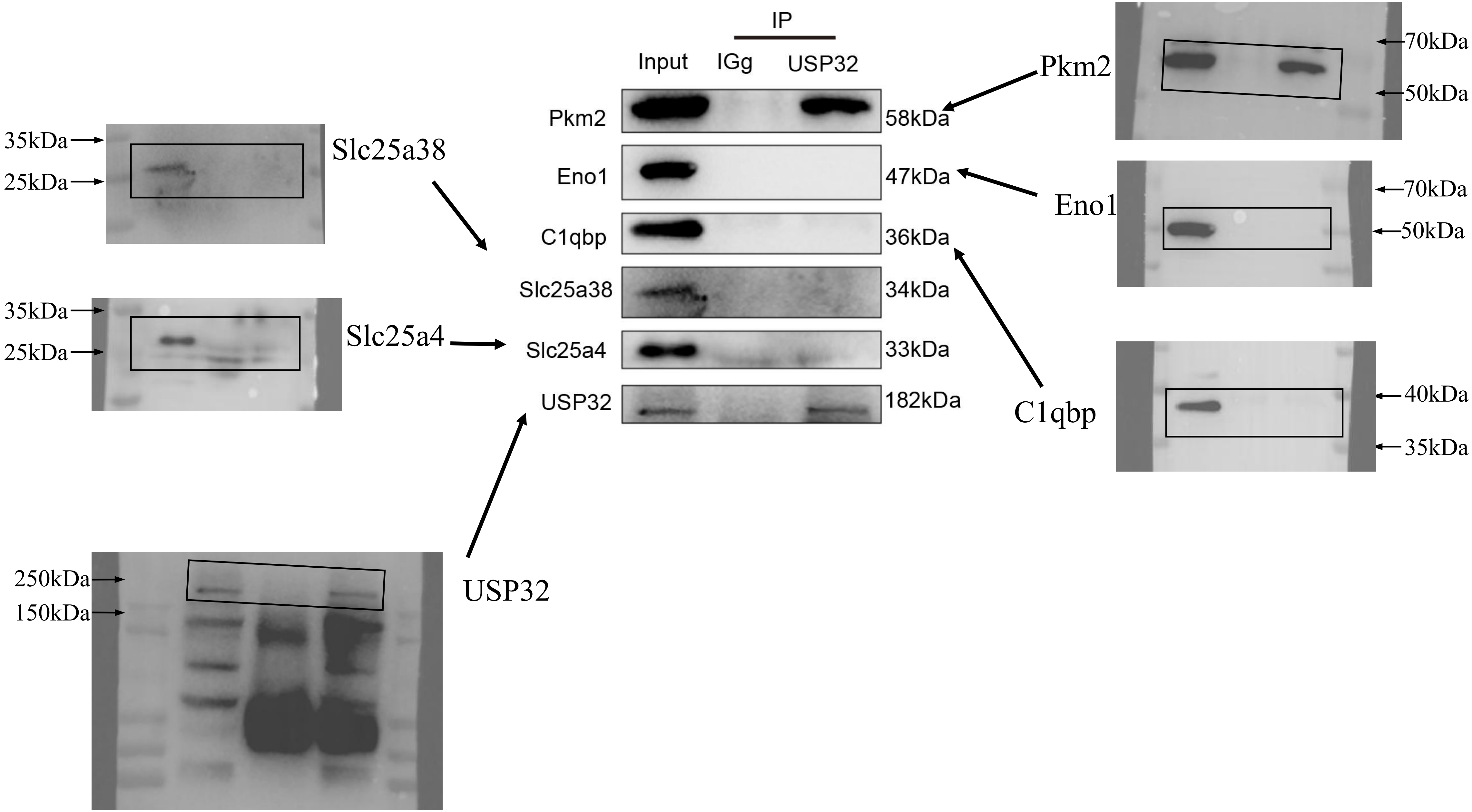
IP
Input
IGg
USP32
Pkm2
58kDa
Eno1
47kDa
C1qbp
36kDa
Slc25a38
34kDa
Slc25a4
33kDa
USP32
182kDa
35kDa
25kDa
Slc25a38
35kDa
25kDa
Slc25a4
250kDa
150kDa
USP32
Pkm2
70kDa
50kDa
Eno1
70kDa
50kDa
C1qbp
40kDa
35kDa

Figure 4D

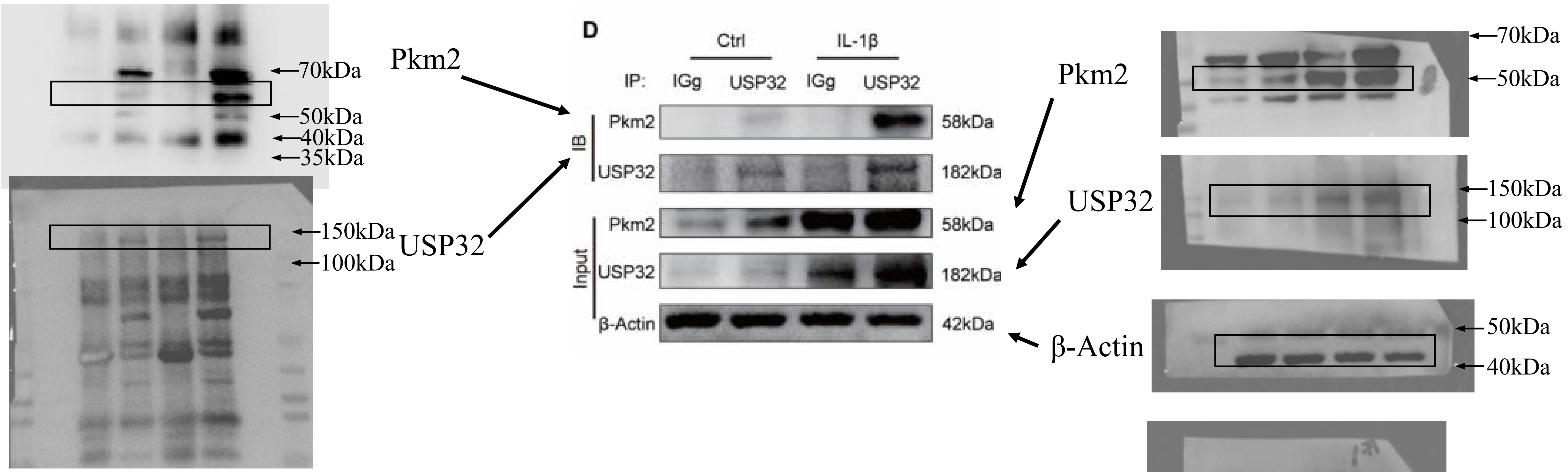

Figure 4E

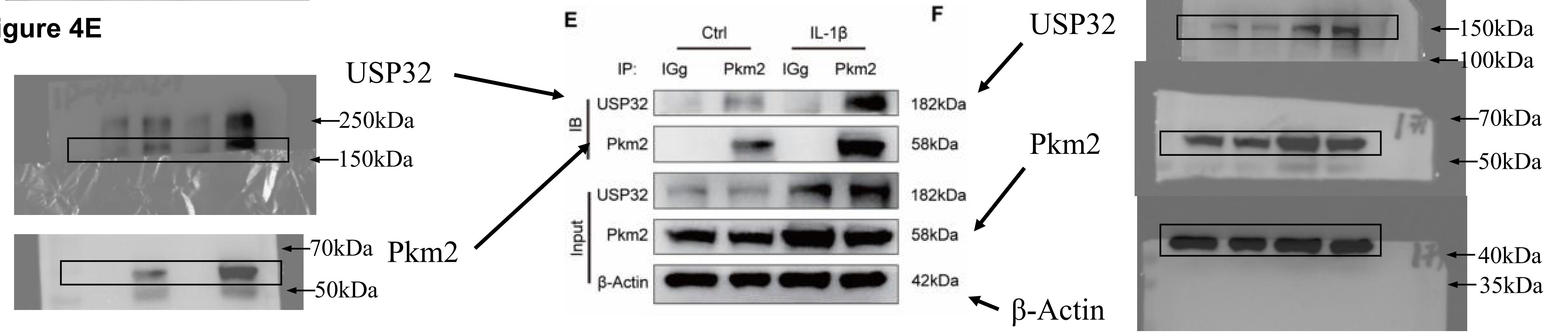

Figure 4I

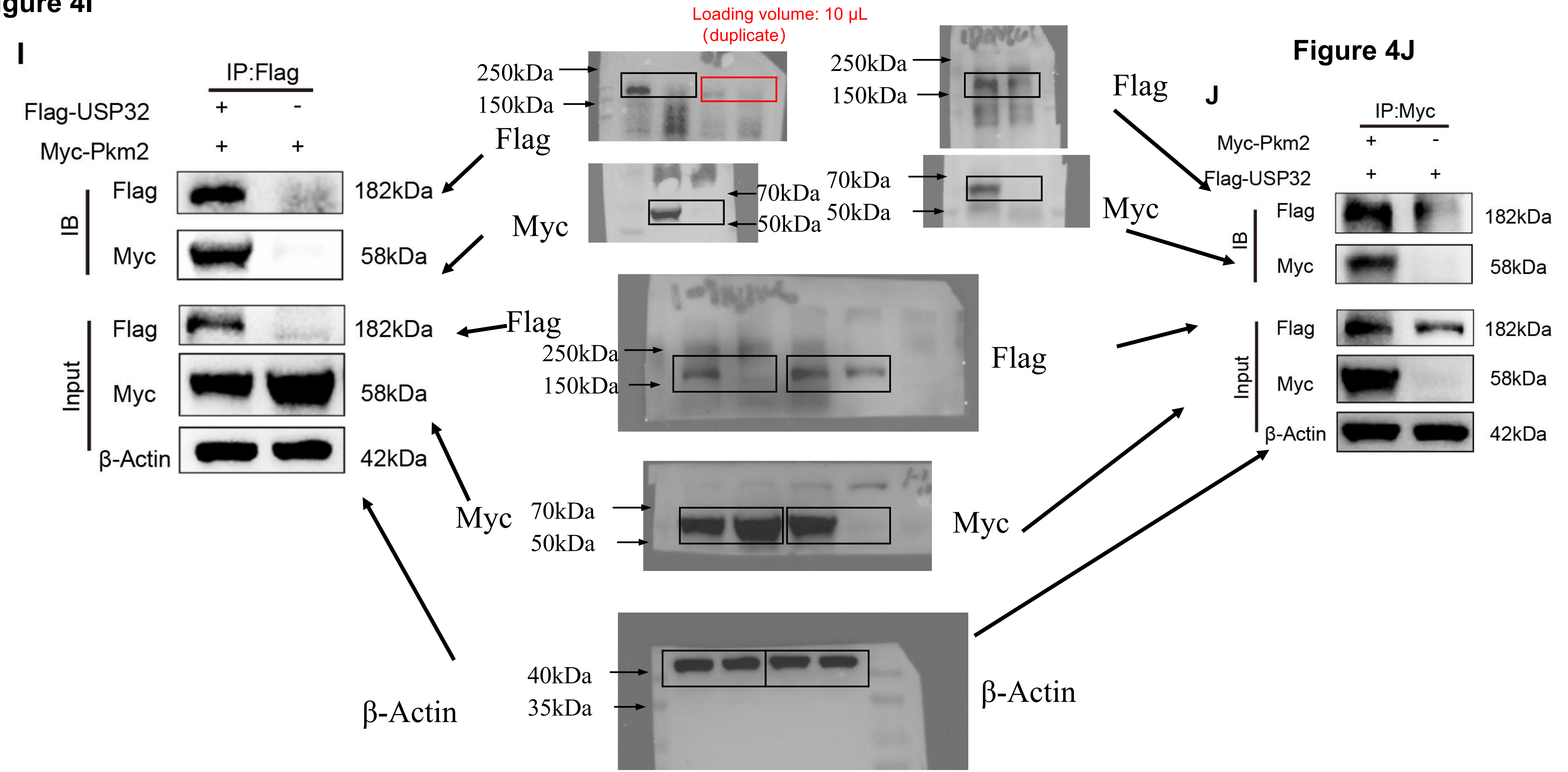

Loading volume: 10 μL
(duplicate)
Figure 4J
I
IP:Flag
Flag-USP32
+
-
Myc-Pkm2
+
+
IB
Flag
182kDa
Myc
58kDa
Input
Flag
182kDa
Myc
58kDa
β-Actin
42kDa
250kDa
150kDa
Flag
Myc
70kDa
50kDa
250kDa
150kDa
Flag
70kDa
50kDa
Myc
250kDa
150kDa
Flag
Myc
70kDa
50kDa
Myc
β-Actin
40kDa
35kDa
β-Actin
J
IP:Myc
Myc-Pkm2
+
-
Flag-USP32
+
+
IB
Flag
182kDa
Myc
58kDa
Input
Flag
182kDa
Myc
58kDa
β-Actin
42kDa

Figure 4M

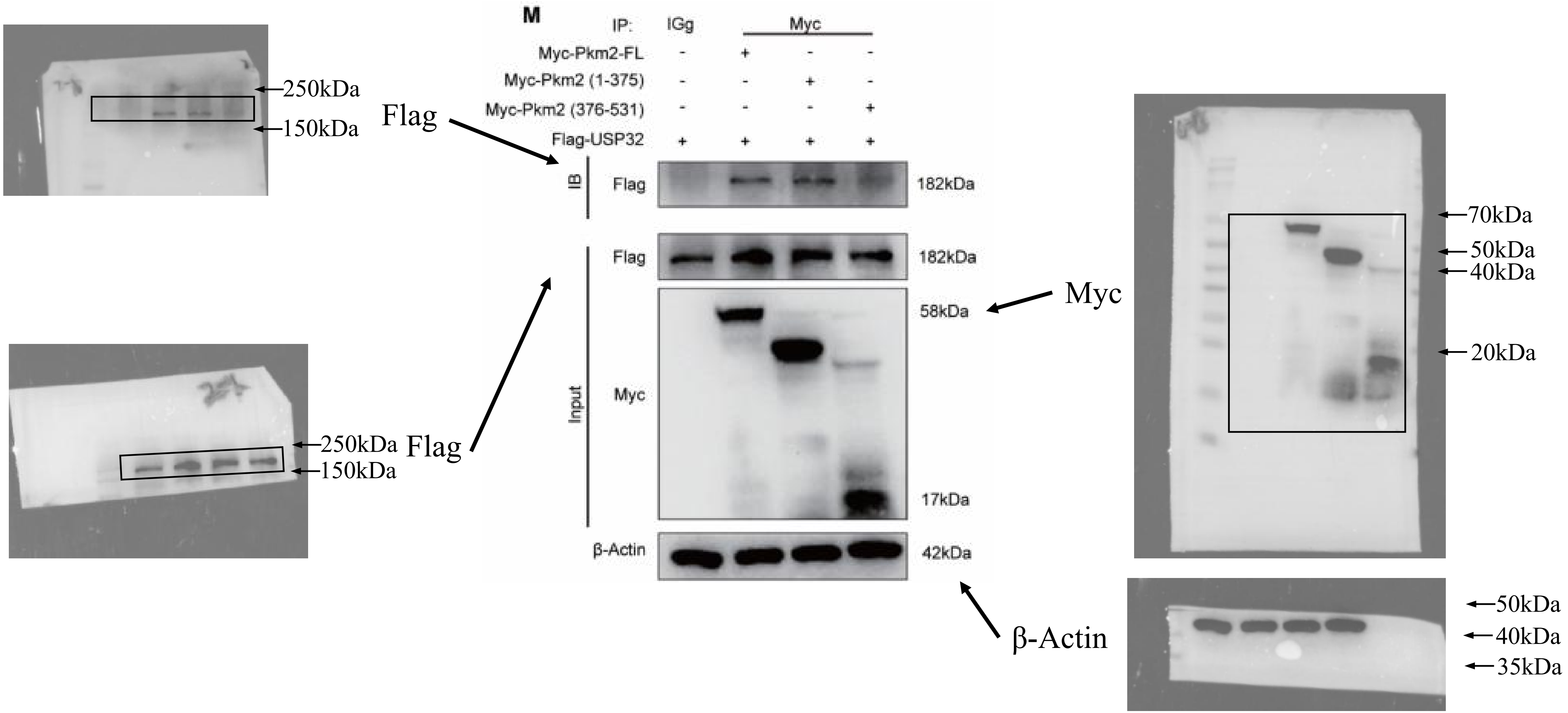
250kDa
150kDa
Flag
250kDa
150kDa
Flag
M
IP:
IGg
Myc
Myc-Pkm2-FL
Myc-Pkm2 (1-375)
Myc-Pkm2 (376-531)
Flag-USP32
IB
Flag
182kDa
Input
Flag
182kDa
Myc
58kDa
17kDa
β-Actin
42kDa
Myc
70kDa
50kDa
40kDa
20kDa
β-Actin
50kDa
40kDa
35kDa

Figure 5A

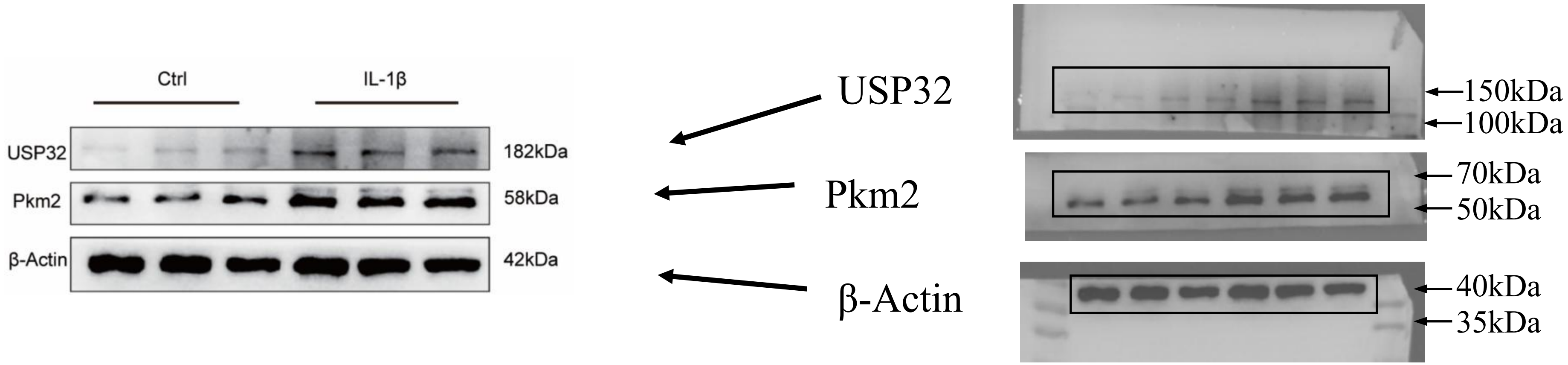

Figure5D

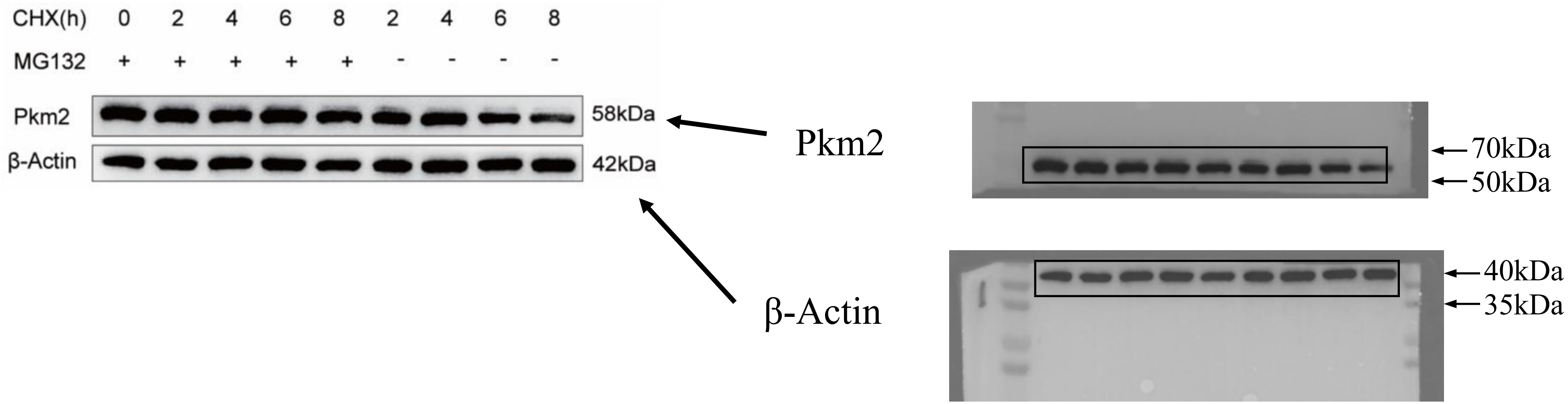

Figure 5F

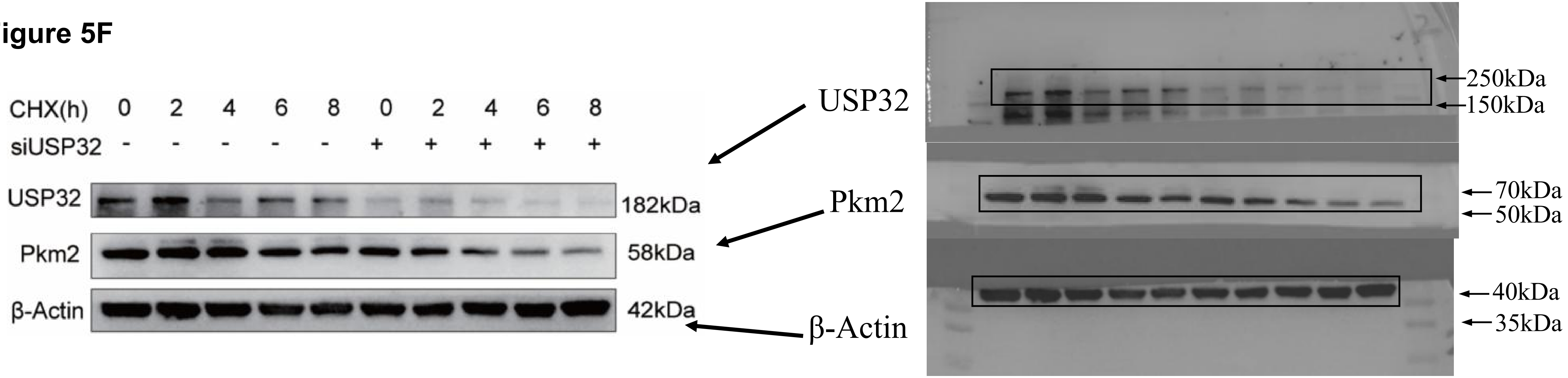

Figure5H

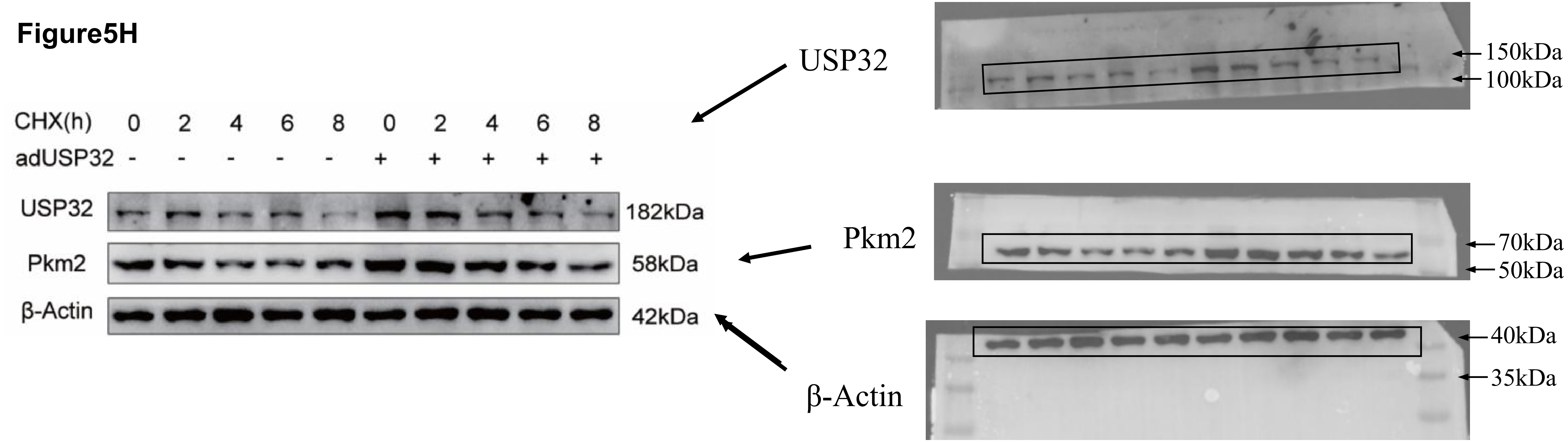

Figure 5J

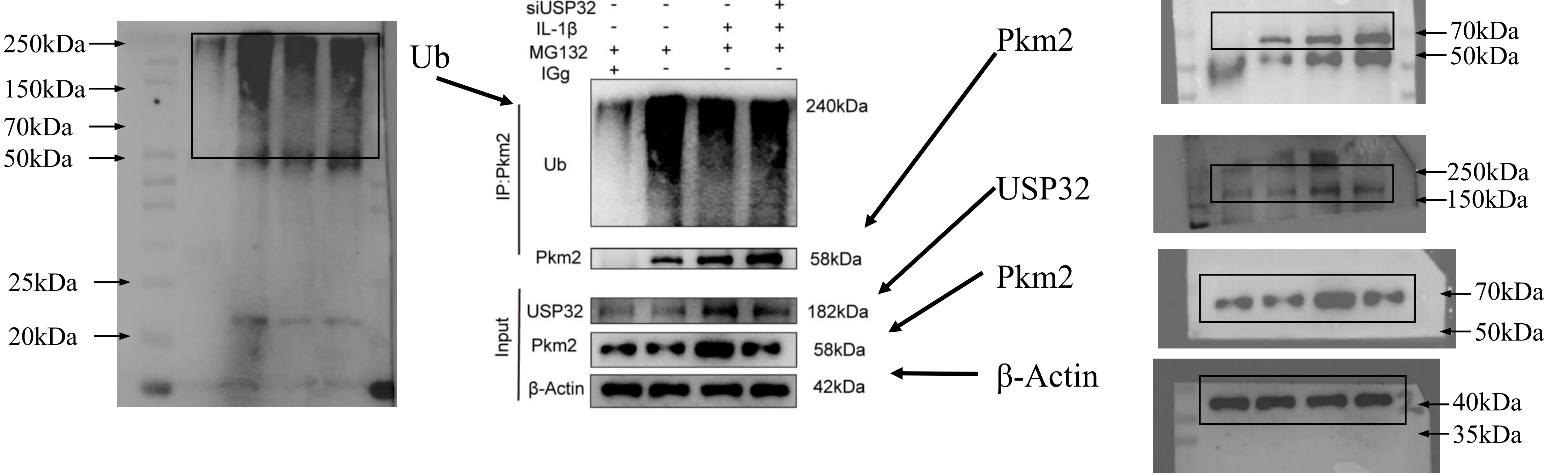

Figure5K

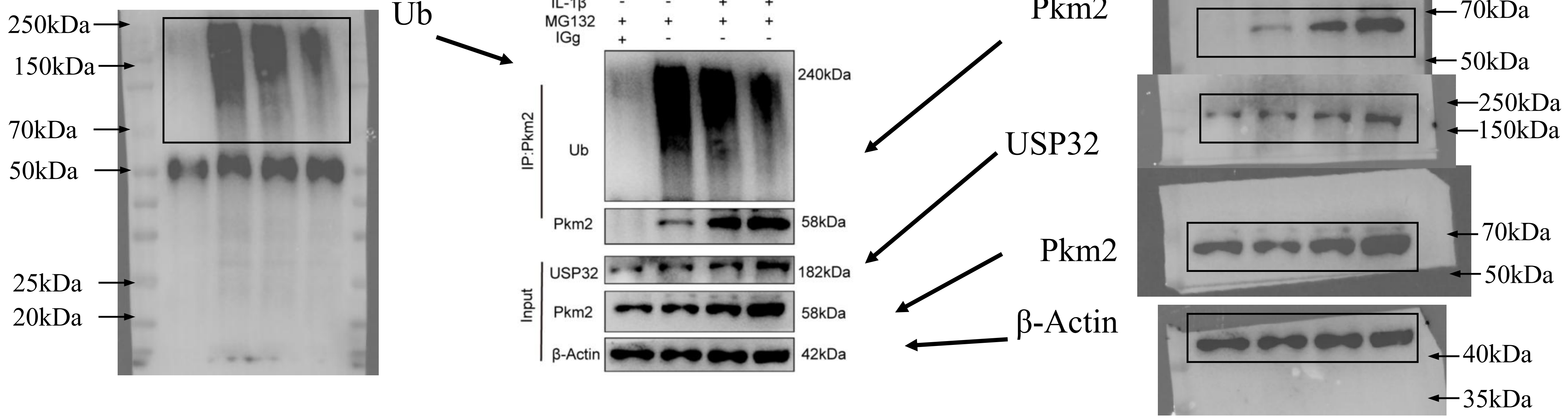

Figure 5L

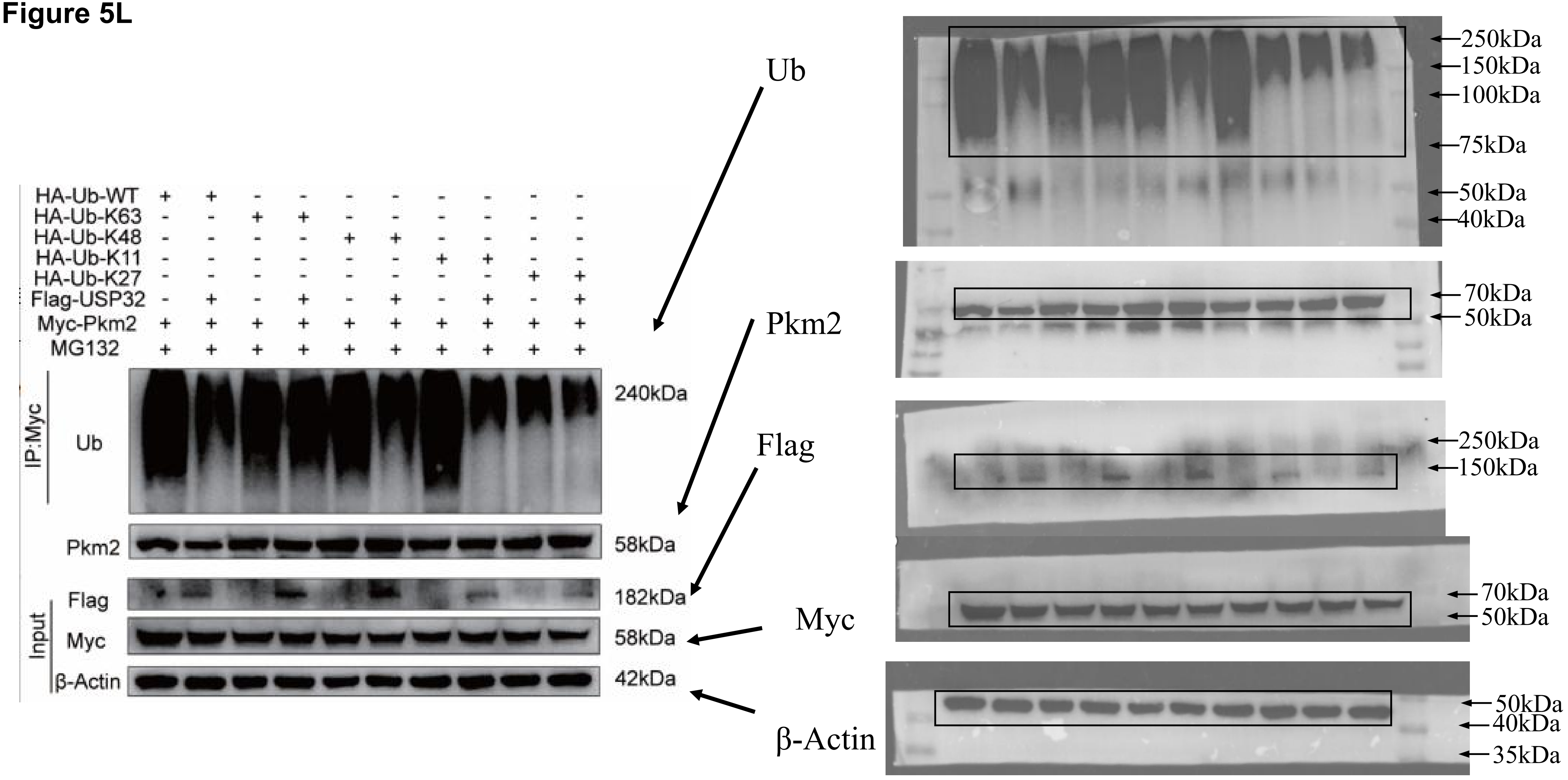
HA-Ub-WT + + - - - - - - - -
HA-Ub-K63 - - + + - - - - - -
HA-Ub-K48 - - - - + + - - - -
HA-Ub-K11 - - - - - - + + - -
HA-Ub-K27 - - - - - - - - + +
Flag-USP32 - + - + - + - + - +
Myc-Pkm2 + + + + + + + + + +
MG132 + + + + + + + + + +
IP:Myc
Ub
240kDa
Pkm2
58kDa
Input
Flag
182kDa
Myc
58kDa
β-Actin
42kDa
Ub
Pkm2
Flag
Myc
β-Actin
250kDa
150kDa
100kDa
75kDa
50kDa
40kDa
70kDa
50kDa
250kDa
150kDa
70kDa
50kDa
50kDa
40kDa
35kDa

Figure 5M

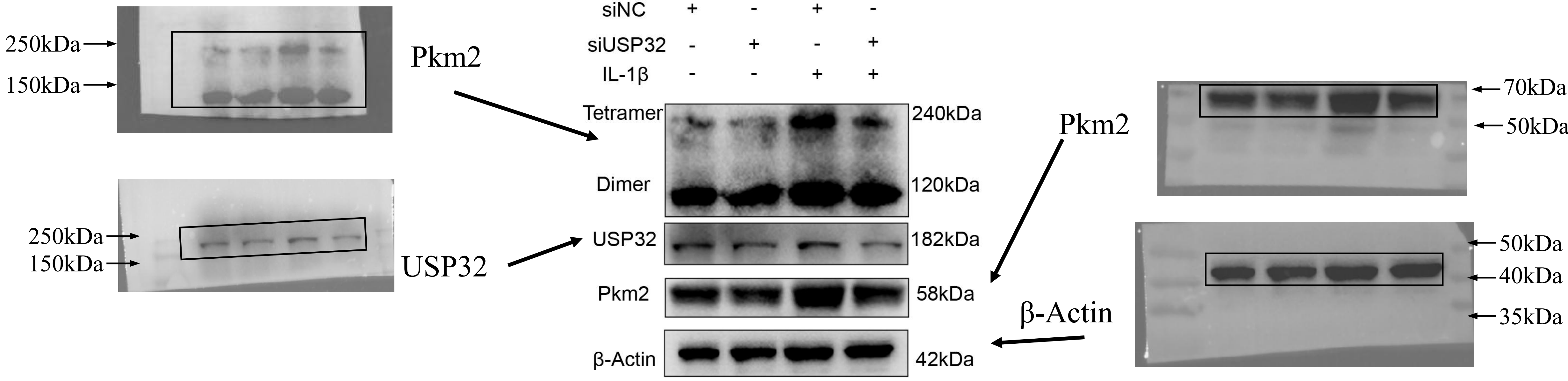

Figure 5O

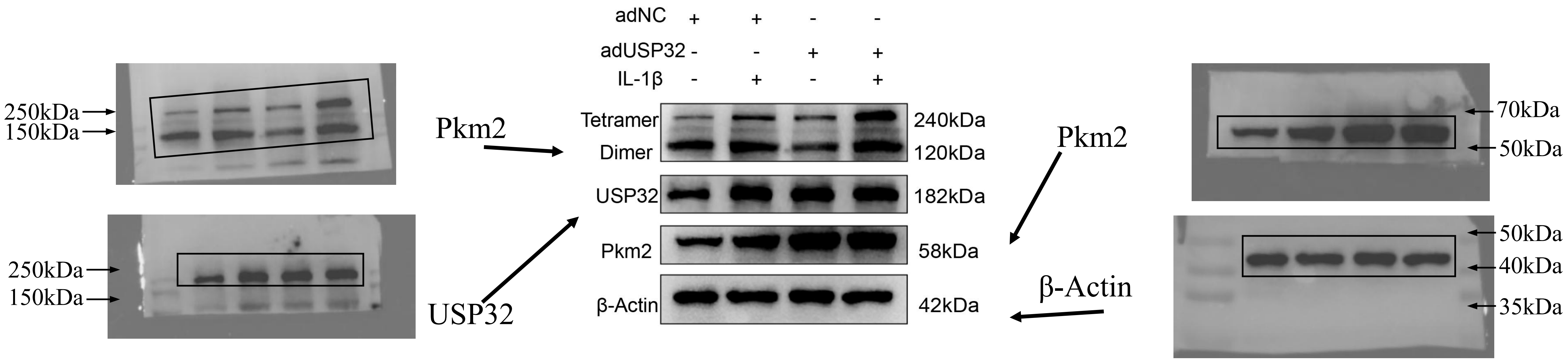

Figure 6A

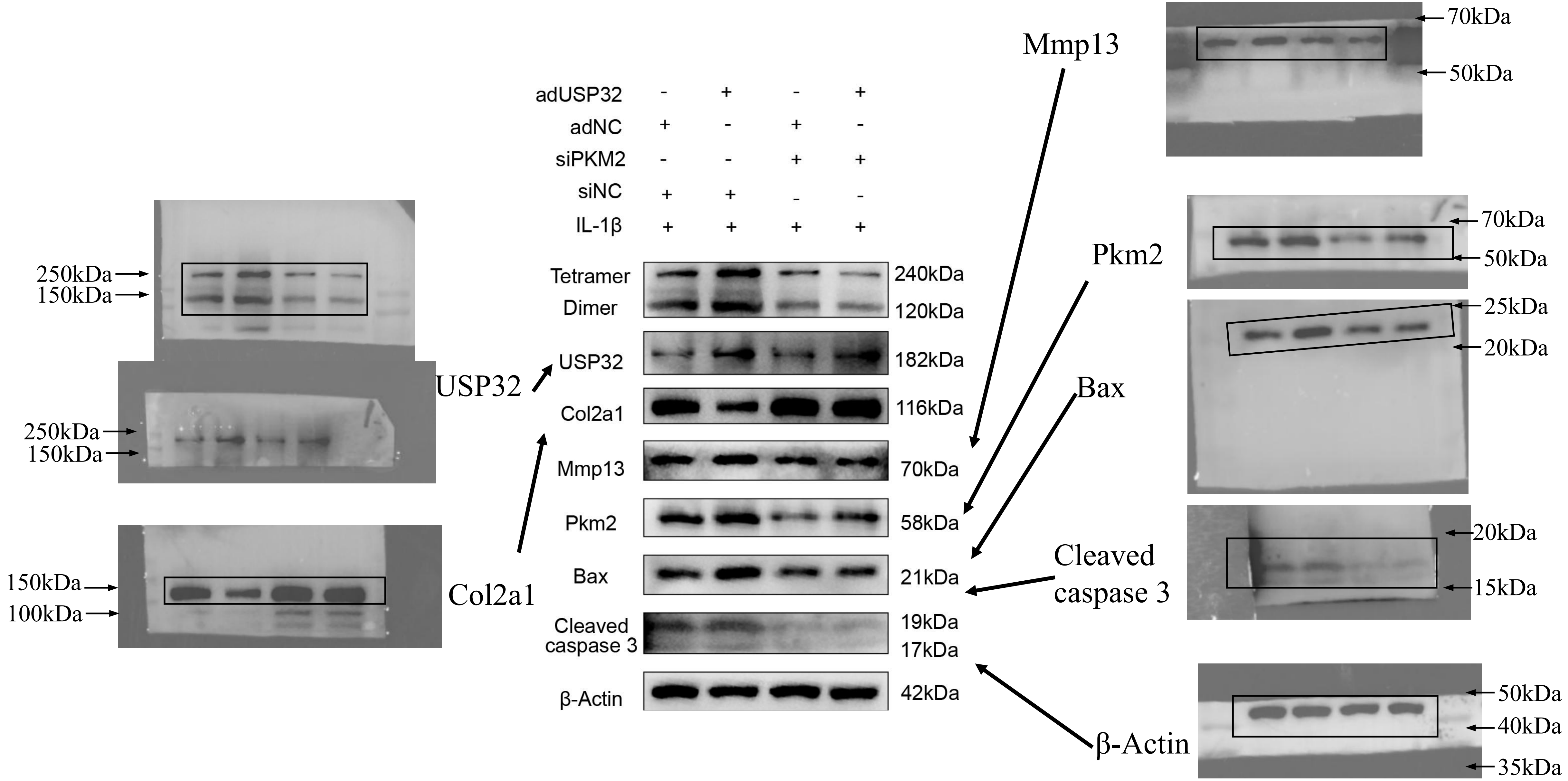
adUSP32 - + - +
adNC + - + -
siPKM2 - - + +
siNC + + - -
IL-1β + + + +
Tetramer
Dimer
240kDa
120kDa
USP32
182kDa
Col2a1
116kDa
Mmp13
70kDa
Pkm2
58kDa
Bax
21kDa
Cleaved caspase 3
19kDa
17kDa
β-Actin
42kDa
250kDa
150kDa
250kDa
150kDa
150kDa
100kDa
USP32
Col2a1
Mmp13
70kDa
50kDa
Pkm2
70kDa
50kDa
25kDa
20kDa
Bax
Cleaved caspase 3
20kDa
15kDa
β-Actin
50kDa
40kDa
35kDa

Figure 7A

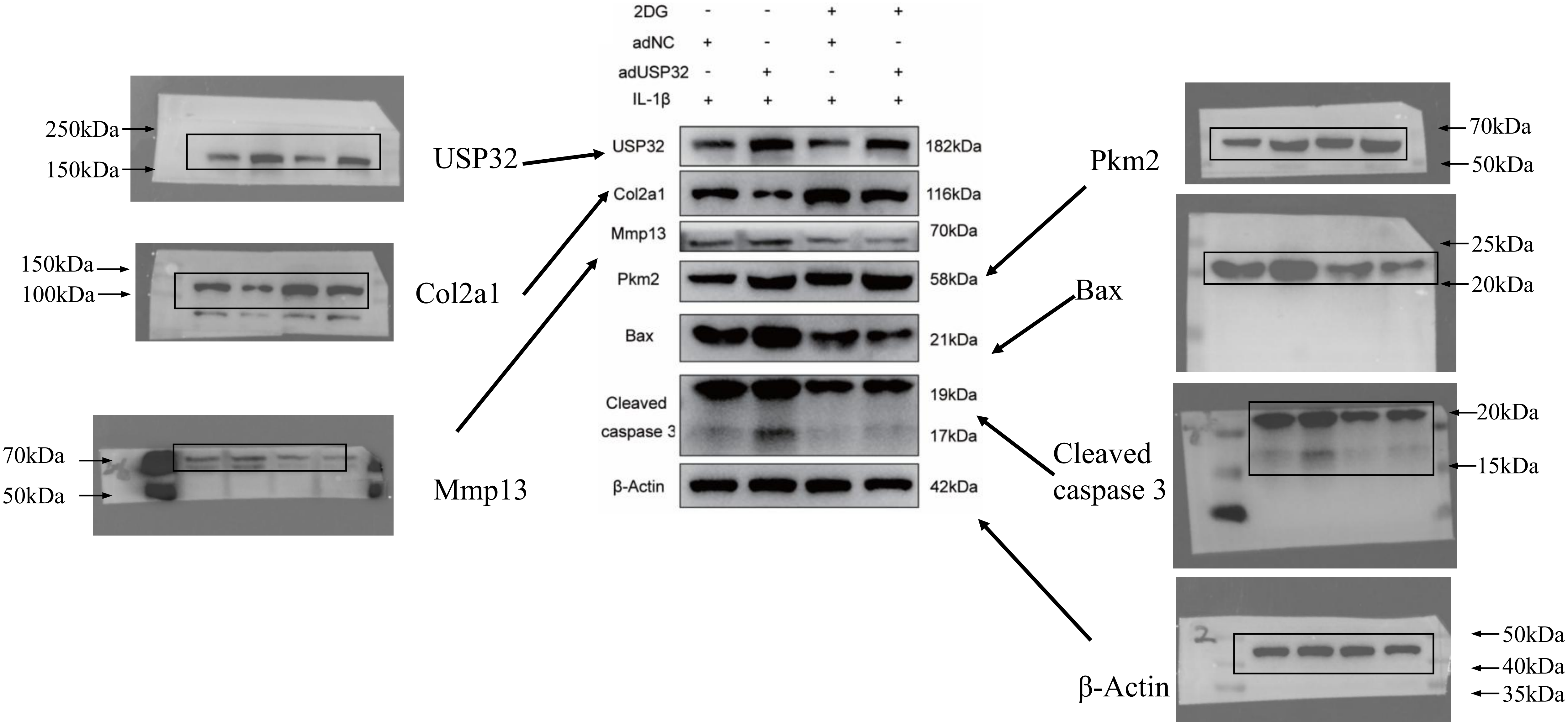

2DG - - + +
adNC + - + -
adUSP32 - + - +
IL-1β + + + +
USP32
182kDa
Col2a1
116kDa
Mmp13
70kDa
Pkm2
58kDa
Bax
21kDa
Cleaved caspase 3
19kDa
17kDa
β-Actin
42kDa
250kDa
150kDa
USP32
150kDa
100kDa
Col2a1
70kDa
50kDa
Mmp13
70kDa
50kDa
Pkm2
25kDa
20kDa
Bax
20kDa
15kDa
Cleaved caspase 3
50kDa
40kDa
35kDa
β-Actin

SFigure4A

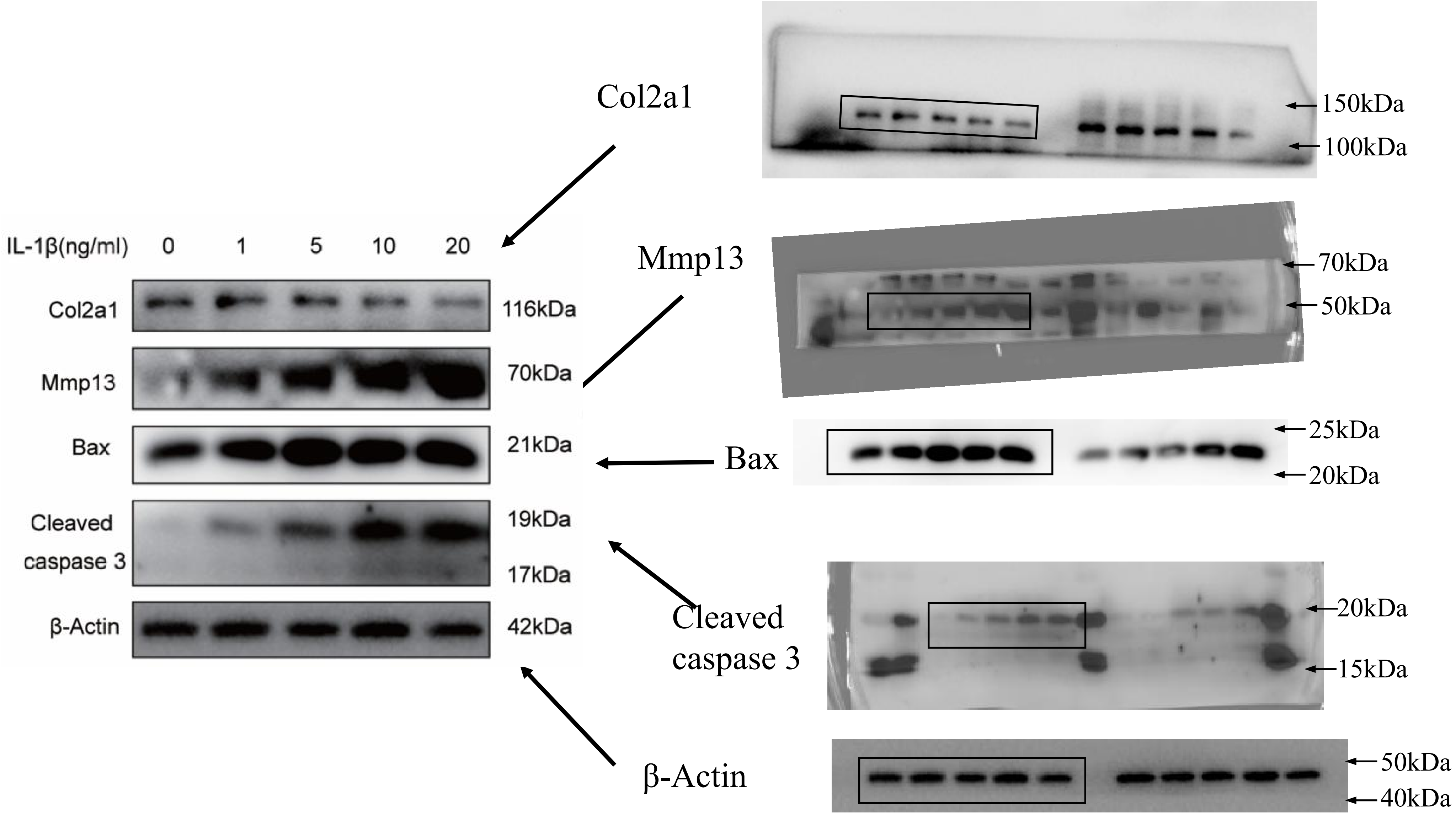
IL-1β(ng/ml) 0 1 5 10 20
Col2a1
116kDa
Mmp13
70kDa
Bax
21kDa
Cleaved caspase 3
19kDa
17kDa
β-Actin
42kDa
Col2a1
150kDa
100kDa
Mmp13
70kDa
50kDa
Bax
25kDa
20kDa
Cleaved caspase 3
20kDa
15kDa
β-Actin
50kDa
40kDa

SFigure5A

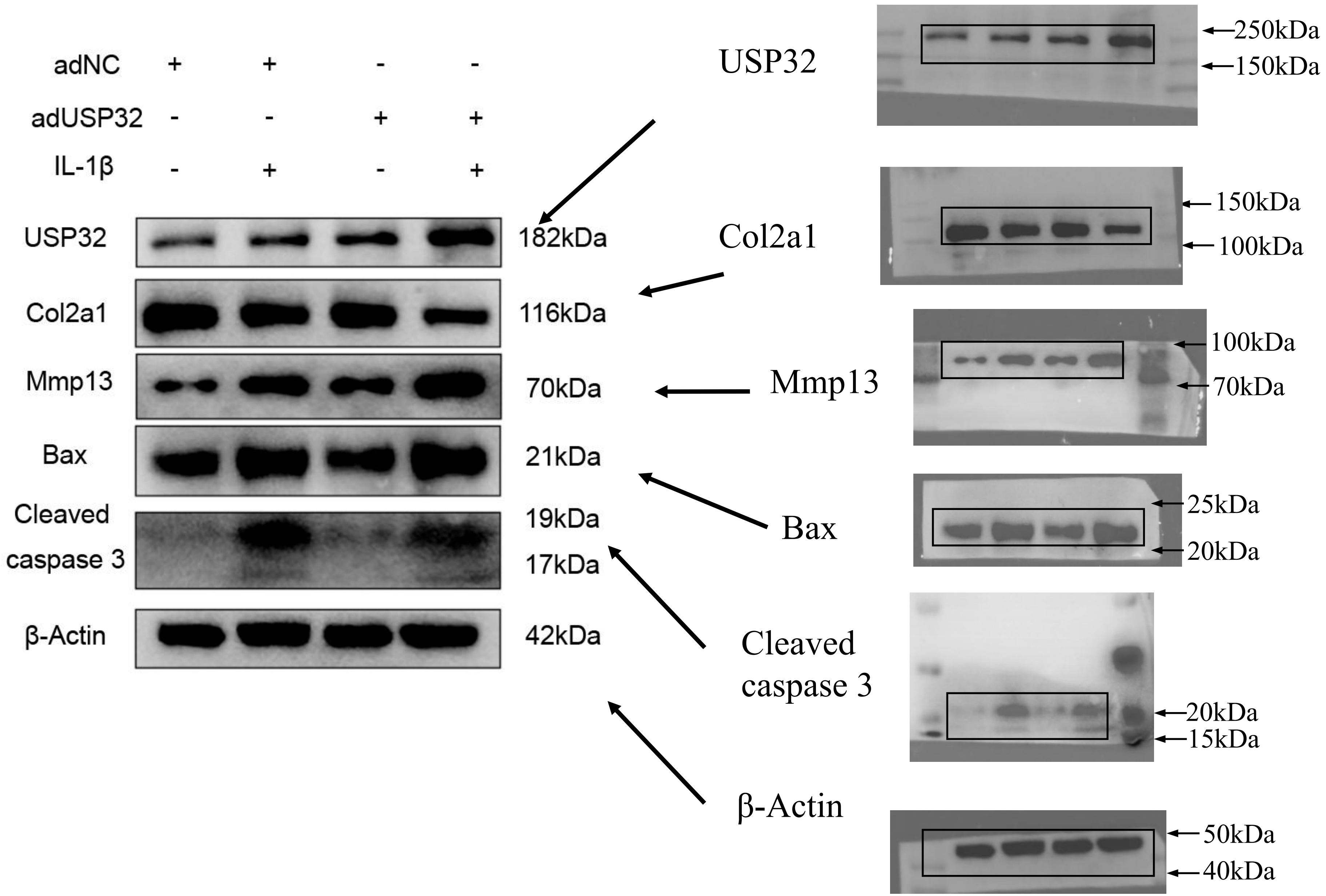
adNC + + - -
adUSP32 - - + +
IL-1β - + - +
USP32
182kDa
Col2a1
116kDa
Mmp13
70kDa
Bax
21kDa
Cleaved caspase 3
19kDa
17kDa
β-Actin
42kDa
USP32
250kDa
150kDa
Col2a1
150kDa
100kDa
Mmp13
100kDa
70kDa
Bax
25kDa
20kDa
Cleaved caspase 3
20kDa
15kDa
β-Actin
50kDa
40kDa

SFigure8B

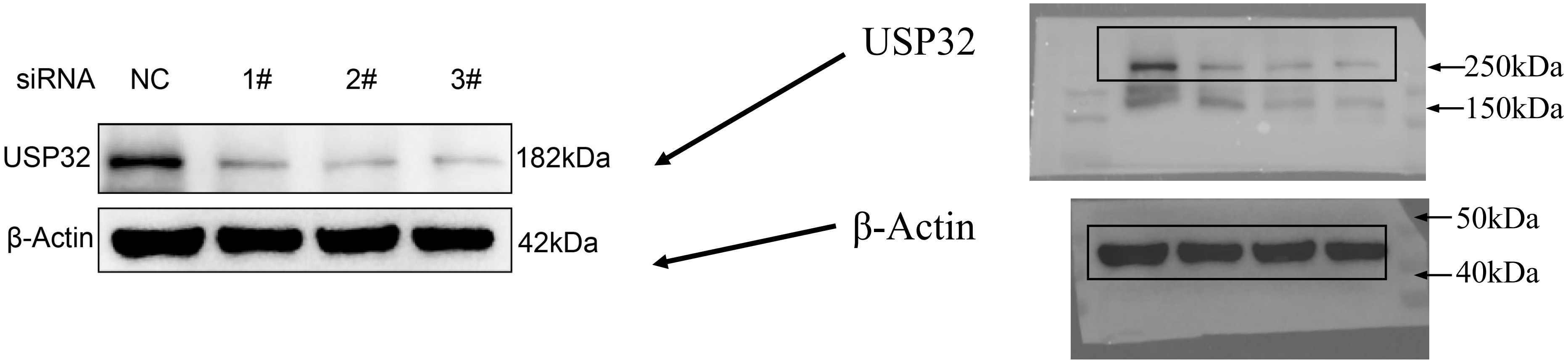

SFigure8D

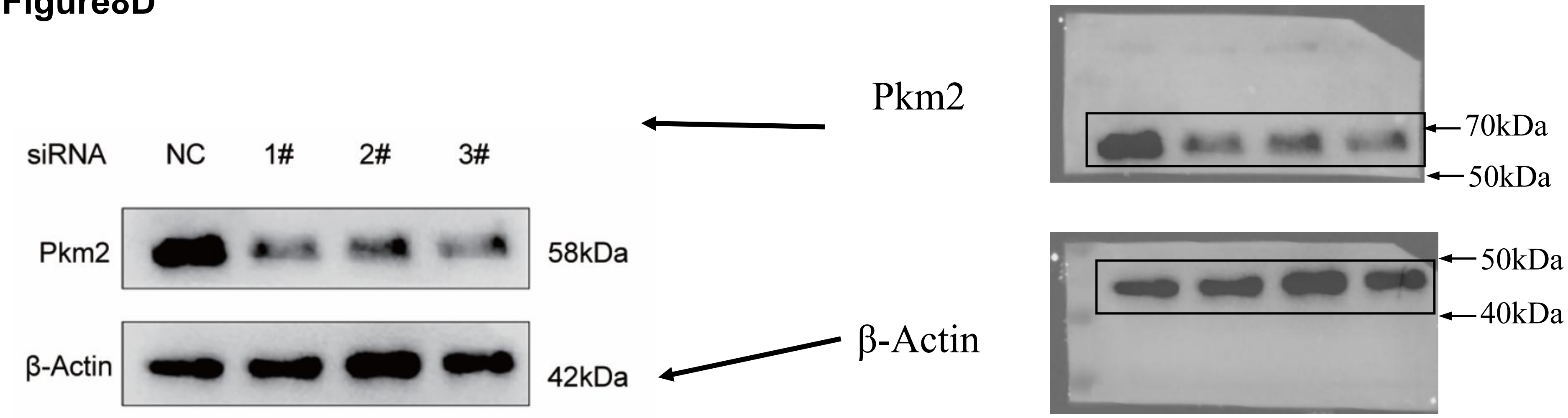

Supplement: Supplementary file 15 — Original Western blots [file 41419_2025_8053_MOESM15_ESM.pdf]
